# Supplementary material for: 3T-VASP: fast ab-initio electrochemical reactor via multi-scale gradient energy minimization
Source: Nat Commun. 2024 Nov 22;15:10140. doi: 10.1038/s41467-024-54453-1 (PMC11584714; doi:10.1038/s41467-024-54453-1)
Supplement: Supplementary file 1 — Supplementary Information [file 41467_2024_54453_MOESM1_ESM.pdf]

Supplementary Information – 3T-VASP: Fast Ab-initio Electrochemical Reactor via Multi-Scale Gradient Energy Minimization

Jonathan P. Mailoa,[1,2,3*] Xin Li,[3] Shengyu Zhang[4*]

1) College of Computer Science and Artificial Intelligence, Wenzhou University, Wenzhou, Zhejiang 325035, China
2) Wenzhou University Artificial Intelligence and Advanced Manufacturing Institute, Wenzhou, Zhejiang 325100, China
3) Tencent Quantum Laboratory, Tencent, Shenzhen, Guangdong 518052, China
4) Tencent Quantum Laboratory, Tencent, Hong Kong SAR, China

[*] corresponding author: jpmailoa@alum.mit.edu, shengyzhang@tencent.com

3T Structure Transformation Modes

In the main text, we have described 6 structure transformation modes utilized in our work: $\mathrm{T_{xyz}}$, $\mathrm{A_m}$, $\mathrm{R_m}$, $\mathrm{T_m}$, $\mathrm{R_M}$ and $\mathrm{T_M}$. $\mathrm{T_{xyz}}$, $\mathrm{T_m}$, and $\mathrm{T_M}$ are just simple Cartesian coordinate translation functions:

$$\mathrm{T_{xyz}}\left(\vec{\mathbf{r}}_\mathbf{i}, \vec{\boldsymbol{\theta}}_{\mathbf{T_{xyz}}}\right) = \vec{\mathbf{r}}_\mathbf{i} + \vec{\boldsymbol{\theta}}_{\mathbf{T_{xyz}},\mathbf{i}} \tag{1}$$

$$\mathrm{T_m}\left(\vec{\mathbf{r}}_\mathbf{i}, \vec{\boldsymbol{\theta}}_{\mathbf{T_m}}\right) = \begin{cases} \vec{\mathbf{r}}_\mathbf{i} + \vec{\boldsymbol{\theta}}_{\mathbf{T_m},\mathbf{j}} & \text{if i} \in \text{micro} - \text{group j} \\ \vec{\mathbf{r}}_\mathbf{i} & \text{otherwise} \end{cases} \tag{2}$$

$$\mathrm{T_M}\left(\vec{\mathbf{r}}_\mathbf{i}, \vec{\boldsymbol{\theta}}_{\mathbf{T_M}}\right) = \begin{cases} \vec{\mathbf{r}}_\mathbf{i} + \vec{\boldsymbol{\theta}}_{\mathbf{T_M},\mathbf{k}} & \text{if i} \in \text{macro} - \text{group k} \\ \vec{\mathbf{r}}_\mathbf{i} & \text{otherwise} \end{cases} \tag{3}$$

with i, j, and k representing individual atom, micro-group, and macro-group indices respectively. The shapes of these parameters are $\mathrm{n_i} \times 3$ (for $\vec{\boldsymbol{\theta}}_{\mathrm{T_{xyz}}}$), $\mathrm{n_m} \times 3$ (for $\vec{\boldsymbol{\theta}}_{\mathrm{T_m}}$), and $\mathrm{n_M} \times 3$ (for $\vec{\boldsymbol{\theta}}_{\mathrm{T_M}}$) with $\mathrm{n_i}$, $\mathrm{n_m}$, and $\mathrm{n_M}$ representing the total number of atoms, micro-groups, and macro-groups.

$\mathrm{A_m}$ is a micro-group sidechain rotation function. In this work, we only allow this transformation to be done on micro-group which is connected to only one other micro-group through a single rotatable bond. Suppose that an atom B in a sidechain micro-group is connected to an atom A in a neighboring micro-group through a rotatable bond. For every atom i (with coordinates $\vec{\mathbf{r}}_\mathrm{i}$) within the sidechain micro-group, we perform the following A–B axis rotation:

$$\vec{\mathbf{U}} = \vec{\mathbf{r}}_\mathrm{B} - \vec{\mathbf{r}}_\mathrm{A} \tag{4}$$

$$\vec{\mathbf{u}} = \vec{\mathbf{U}} / \left|\vec{\mathbf{U}}\right| \tag{5}$$

$$\vec{\mathbf{R}}_\mathrm{i} = \vec{\mathbf{r}}_\mathrm{i} - \vec{\mathbf{r}}_\mathrm{A} \tag{6}$$

$$\vec{\mathbf{Z}}_\mathrm{i} = \left|\vec{\mathbf{R}}_\mathrm{i} \cdot \vec{\mathbf{u}}\right| \vec{\mathbf{u}} \tag{7}$$

$$\vec{\mathbf{x}}_\mathrm{i} = \vec{\mathbf{R}}_\mathrm{i} - \vec{\mathbf{Z}}_\mathrm{i} \tag{8}$$

$$\vec{\mathbf{y}}_\mathbf{i} = \vec{\mathbf{u}} \times \vec{\mathbf{x}}_\mathrm{i} \tag{9}$$

$$\mathrm{A_m}\left(\vec{\mathbf{r}}_\mathrm{i}, \vec{\boldsymbol{\theta}}_{\mathrm{A_m}}\right) = \begin{cases} \vec{\mathbf{r}}_\mathrm{A} + \vec{\mathbf{Z}}_\mathrm{i} + \vec{\mathbf{x}}_\mathrm{i} \cos \theta_{\mathrm{A_m},\mathrm{j}} + \vec{\mathbf{y}}_\mathbf{i} \sin \theta_{\mathrm{A_m},\mathrm{j}} & \text{if i} \in \text{micro} - \text{group j} \\ \vec{\mathbf{r}}_\mathrm{i} & \text{otherwise} \end{cases} \tag{10}$$

The shape of the parameter $\vec{\boldsymbol{\theta}}_{A_m}$ is $n_{A_m} \times 1$, with n_{A_m} representing the total number of sidechain micro-groups with just one rotatable bond connection.

R_m and R_M are three-axis rotation functions centered on either the micro-group or the macro-group centers. For every atom i within the micro-group j (or atom i within macro-group k), we perform:

$$R_a = \begin{bmatrix} \cos\theta_{R_m,j,0} & -\sin\theta_{R_m,j,0} & 0 \\ \sin\theta_{R_m,j,0} & \cos\theta_{R_m,j,0} & 0 \\ 0 & 0 & 1 \end{bmatrix} \tag{11}$$

$$R_b = \begin{bmatrix} \cos\theta_{R_m,j,1} & 0 & \sin\theta_{R_m,j,1} \\ 0 & 1 & 0 \\ -\sin\theta_{R_m,j,1} & 0 & \cos\theta_{R_m,j,1} \end{bmatrix} \tag{12}$$

$$R_c = \begin{bmatrix} 1 & 0 & 0 \\ 0 & \cos\theta_{R_m,j,2} & -\sin\theta_{R_m,j,2} \\ 0 & \sin\theta_{R_m,j,2} & \cos\theta_{R_m,j,2} \end{bmatrix} \tag{13}$$

$$\vec{\mathbf{r}}_C = \frac{1}{n_j}\sum_i \vec{\mathbf{r}}_i \quad \text{for i} \in \text{micro} - \text{group j} \tag{14}$$

$$R_m\left(\vec{\mathbf{r}}_i, \vec{\boldsymbol{\theta}}_{R_m}\right) = \begin{cases} \vec{\mathbf{r}}_C + R_a R_b R_c(\vec{\mathbf{r}}_i - \vec{\mathbf{r}}_C) & \text{if i} \in \text{micro} - \text{group j} \\ \vec{\mathbf{r}}_i & \text{otherwise} \end{cases} \tag{15}$$

The equations above are written for R_m rotation of micro-group j. For R_M rotation of macro-group k, simply substitute m with M and micro-group j with macro-group k. The shapes of these parameters are $n_m \times 3$ (for $\vec{\boldsymbol{\theta}}_{R_m}$) and $n_M \times 3$ (for $\vec{\boldsymbol{\theta}}_{R_M}$) respectively.

TEP4PBA$^+$ Relaxation on $FAPbI_3$ FA$^+$ Vacancy Defect Surface

The TEP4PBA$^+$ cation molecule is relaxed on the FAPbI3 surface with FA+ vacancy defect, starting from three different initial TEP4PBA$^+$ conformations, for both standard DFT relaxation and 3T relaxation approaches. The initial structure for 3T-FF was constructed by placing the cation with random rotation and translation within a cubic space of 10×10×10 Å^3 centred directly above the defect site using packmol, with the bottom of the cube being 1 Å above the highest perovskite atom. The binding energies are available in the main text, while the resulting relaxed geometries are shown in **Supplementary Figure 1** below.

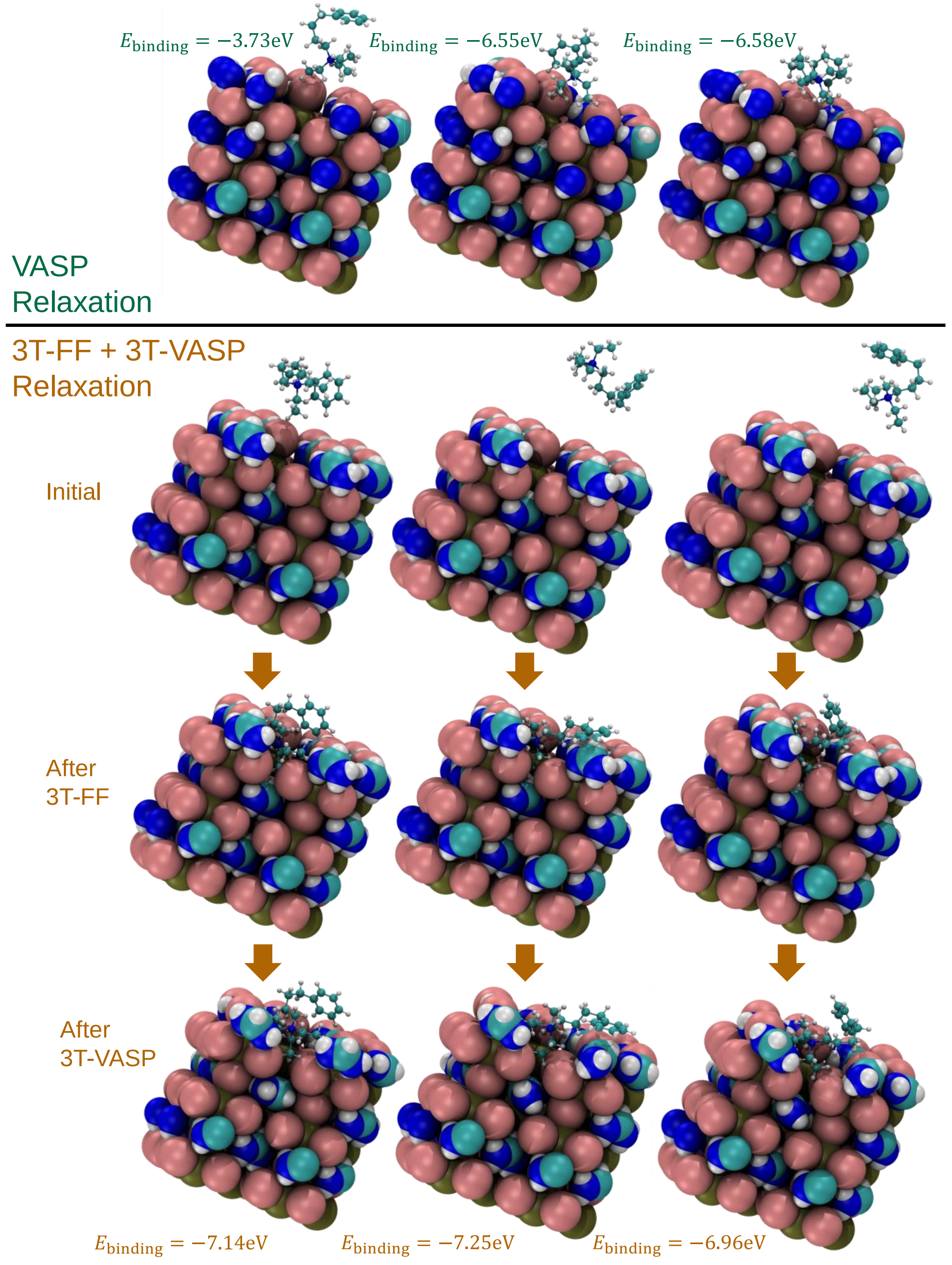

Supplementary Figure 1 | Final $FAPbI_3$–TEP4PBA structures for both standard DFT relaxation and 3T relaxation with different initial TEP4PBA positions. The three standard DFT relaxations based on conjugate gradient atomic forces fail to find TEP4PBA conformation entering the vacancy defect site, while the three 3T structures all embed TEP4PBA deep into the FA^+ vacancy defect sites with larger DFT binding energy. Relatively different 3T TEP4PBA final poses indicate several deep local energy minimums. For 3T relaxation, we show the initial structure (prior to 3T-FF), the structure after 3T-FF relaxation, and the structure after 3T-VASP relaxation.

Additional Perovskite Surface Cation Passivation Baseline Comparison using CREST + VASP Relaxation

It is also possible to obtain better passivation structure for single TE4PBA cation passivation on the $FAPbI_3$ surface vacancy defect by first performing Conformer-Rotamer Ensemble Sampling Tool (CREST)-based[9] TE4PBA conformation search on the $FAPbI_3$ surface before performing standard VASP relaxation. Multiple conformers for the large floppy TE4PBA cation have been generated in the presence of defective $FAPbI_3$ perovskite surface using CREST. The TE4PBA cation is very large and floppy, so the conformation search took a long time and we had to stop it after 5 days of conformation search.

To enhance the sampling of molecular structure, we initially conducted molecular dynamics simulations using xTB under the GFN0-xTB framework.[10,11] The simulations were performed at a temperature of 600 K with a time step of 1 fs for a total duration of 10 ps. Structures were saved every 5 fs, and the parameters hmass=1 and shake=0 were set, resulting in a total of 2000 sampled structures. These 2000 molecular structures were then subjected to batch optimization using the mdopt function of CREST under the GFN0-xTB level of theory. Subsequently, the optimized structures were processed through isostat of Molclus[12] for duplicate removal and energy ranking, with an energy duplicate threshold of 0.5 kcal/mol and a structural duplicate threshold of 0.5 Å, without calculating the Boltzmann distribution ratio. Following clustering, 347 structures remained. The top 100 structures were further optimized using the mdopt function of CREST under the GFN1-xTB[10,11] level of theory. Using the same configuration, isostat was again employed for duplicate removal and energy ranking for post-optimized structures. This step did not eliminate any structures.

Three TE4PBA conformation structures with the lowest CREST energies are chosen. Because CREST also distorts the perovskite surface structure (even though we have set the CREST settings to freeze the surface atoms), we then transfer the CREST-generated conformer molecule coordinates onto the original defective perovskite surface on top of the A-site vacancy defect. An additional standard VASP relaxation is then performed. We can see from the result in main text **Figure 2f** that

while CREST + VASP is better than the baseline standard VASP relaxation, CREST + VASP results are still worse than 3T-VASP (in addition to taking significantly more DFT relaxation steps and prior lengthy cation conformation search). The end structures of the CREST + VASP relaxation structures are shown in **Supplementary Figure 2** below.

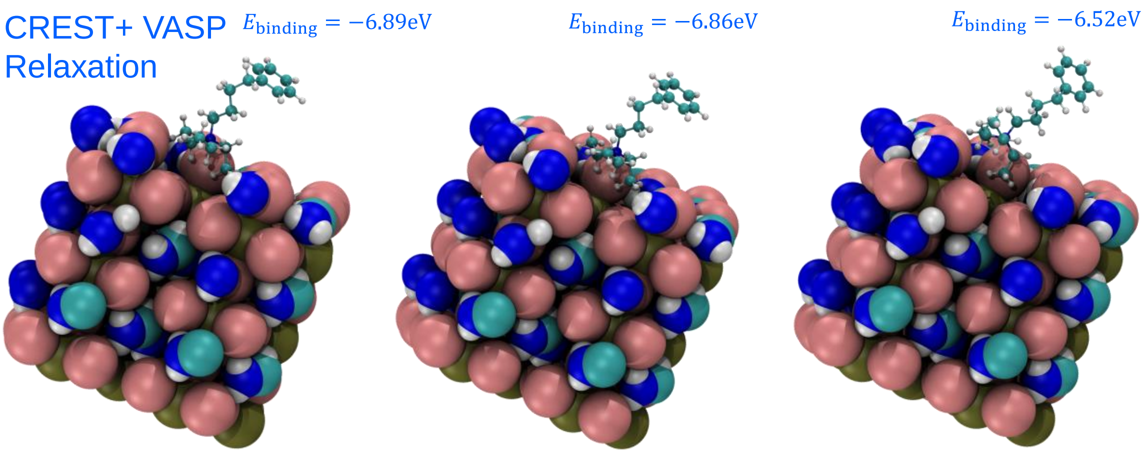

Supplementary Figure 2 | TE4PBA cation passivation structure on $FAPbI_3$ defect site after CREST conformation search followed by VASP relaxation. These cations can slightly enter the defect sites, although the binding energies are still higher than the deeply embedded cation structures found by 3T relaxation.

Additional Perovskite Surface Cation Passivation Baseline Comparison using FF + VASP Relaxation

It is also possible to utilize our force field pipeline to generate a better baseline for the VASP relaxation without using 3T. We first take the 3 initial structures we previously utilized for standard DFT relaxation and run them through 5×200 steps of FF relaxation steps. This is done using our PyTorch optimization pipeline while turning off the 3T capabilities (this becomes a standard per-atom relaxation, and not a 3T multi-scale relaxation). The resulting structure can then be minimized using VASP. The result using this improved baseline approach is promising, with the TE4PBA cation becoming deeply embedded into the defect site, with energy levels equivalent to that produced using 3T-VASP. However, the FF+VASP approach still requires significantly more DFT steps compared to 3T-VASP (main text **Figure 2f**). The resulting FF+VASP relaxation structures are shown in **Supplementary Figure 3** below.

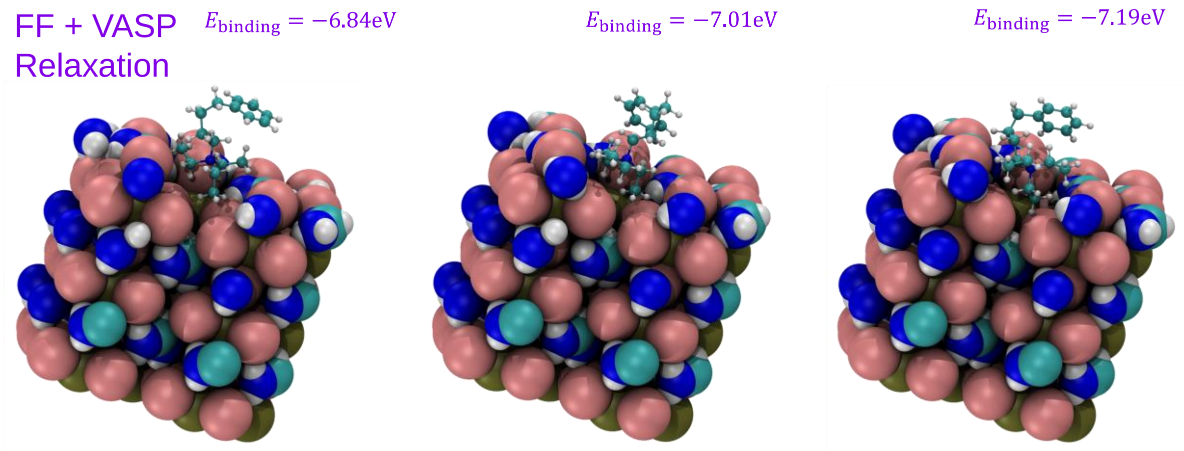

Supplementary Figure 3 | TE4PBA cation passivation structure on $FAPbI_3$ defect site after we use our FF per-atom relaxation followed by VASP relaxation. These cations are deeply embedded into the defect site just like the 3T-VASP relaxation version, although they still require significantly more DFT steps than 3T-VASP relaxation.

Perovskite Surface Passivation Transition State and Energy Barrier Analysis

We further perform transition state analysis and determine the energy barrier of our $FAPbI_3$ A-site vacancy defect passivation using TE4PBA cation, using the trajectory generated by our 3T minimization. The cation embedding is first observed during the 3T-FF cycle, so we extracted a snapshot from the end of 3T-FF (post-snapshot, with the cation already deeply embedded into the defect site), a snapshot in the 3T-FF trajectory where the cation has not been embedded into the defect site yet (pre-snapshot), and a third snapshot with the cation being positioned somewhere in the middle of the pre-snapshot and post-snapshot (mid-snapshot) which is generated by nudged elastic band (NEB). The pre-snapshot and post-snapshot are then relaxed using standard VASP relaxation, as we need these snapshots to be in their respective local energy minimums. We then perform NEB using VASP which includes VTST (Transition State Tools for VASP) tools on the three structures (relaxed pre-snapshot, mid-snapshot, and relaxed post-snapshot). An intermediate saddle-point structure is obtained, with an energy barrier of 0.932 eV higher than the relaxed pre-snapshot.

This intermediate snapshot is then used to perform a transition state analysis. Because the TE4PBA is a very large and floppy molecule, we observe 2 trivial sidechain rotations vibration modes (one $-CH_3$ group and one $-CH_2$ group rotation) in addition to the real transition state vibration mode representing the cation embedding into the defect site. Five individual hydrogen atoms on the sidechains which are located away from the core vibration region of the transition state vibration mode are restricted from moving in the *x* axis to eliminate the trivial sidechain rotations, and only the real transition state vibration mode representing the molecule embedding event remains. We then slightly perturb this cation structure according to the transition state vibration mode in both directions (toward the relaxed pre-snapshot and the relaxed post-snapshot directions) and relax the structure using standard VASP relaxation, producing two trajectories where the transition state TE4PBA cation moves back in the direction of either the relaxed pre-snapshot or to the relaxed post-snapshot. Note the structure and energy gap between the relaxed pre/post-snapshots and the structures relaxed from

the NEB transition state. This indicates that there are additional transition states and energy barriers in the reaction trajectory landscape because the TE4PBA cation is a very large and floppy molecule. We also use this trajectory to plot the energy vs specific cation-perovskite atom distance, as shown in **Supplementary Figure 4** below.

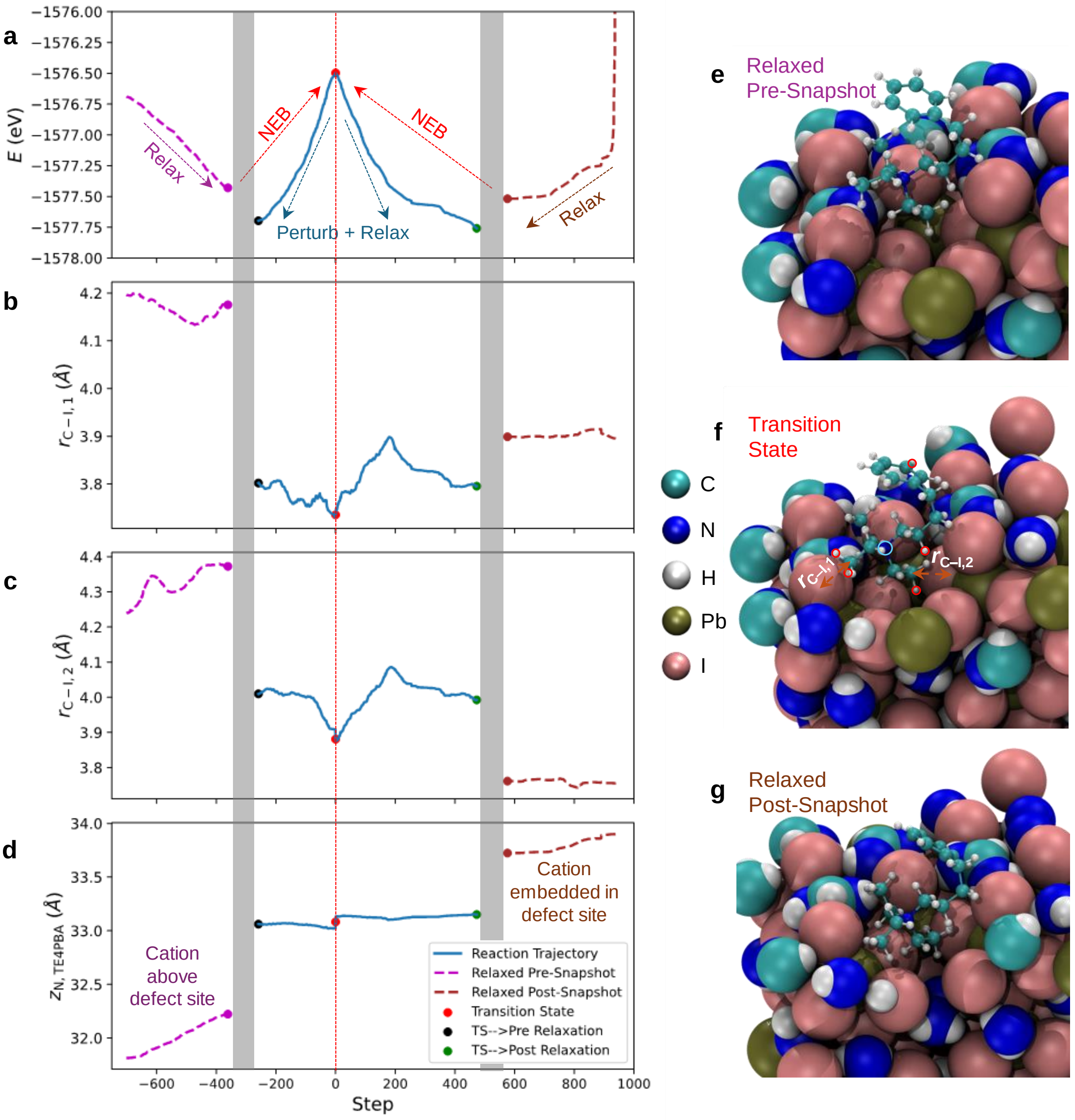

Supplementary Figure 4 | Transition state and energy barrier analysis using nudged elastic band (NEB). The structures are obtained by first relaxing the pre-snapshot and post-snapshots extracted from 3T-FF trajectory using VASP. The transition state structure is then obtained using NEB utilizing the relaxed pre-snapshot and post-snapshot, in addition to a 3T-FF snapshot located in between the pre- and post-snapshots. The transition state structure is perturbed slightly in either direction according to the transition state vibration mode, and further relaxed using VASP to generate additional trajectory in the immediate vicinity of the transition state. We show the structures' DFT energy **(a)**, distance between bonding C and I atoms $r_{C-I,1}$ **(b)** and $r_{C-I,2}$ **(c)**, as well as the *z* axis Cartesian coordinate of TE4PBA nitrogen atom (+*z* axis points into the surface) **(d)** which undergoes a clear step function during the state transition. The relaxed pre-snapshot **(e)**, transition state **(f)**, and relaxed post-snapshot **(g)** are shown. The sidechain H atoms which are restricted from moving along the *x* axis during transition state vibration mode analysis are highlighted using the red circles, while the N atom of TE4PBA is highlighted using the light blue circle. The bonding C and I atoms $r_{C-I,1}$ and $r_{C-I,2}$ are also shown. Source data are provided as a Source Data file.

Electrolyte Molecule Segmentation and 3T Redox Box Setup

The molecules are segmented into micro-groups based on their rotatable bonds as defined by RDKit.[1] Entire molecules are grouped into separate macro-groups. Based on this segmentation criteria, the micro-group segmentation is shown below for the electrolyte input molecules:

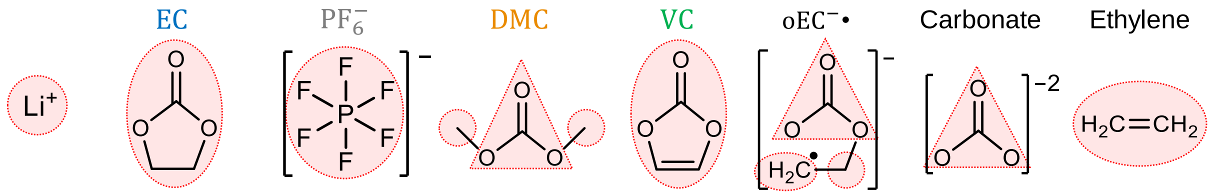

Supplementary Figure 5 | Micro-group segmentation of electrolyte input molecules.

A periodic simulation box is prepared with the size of 14×14×14 Å^3 and n_{Li} lithium atoms are placed within. We then place the desired number of electrolyte molecules inside the remaining space within the box using PackMol[2] (see the main text for the molecule counts) before we start the 3T energy minimization. We perform several cycles of 3T-FF (no chemical reaction possible, only liquid molecule dispersion happens at this stage), followed by several cycles of 3T-VASP (chemical reactions only start happening at this stage).

Modified 3T Classical Force Field Parameters for $\mathrm{oEC}^{-}\bullet$ and CO_3^{2-}

The classical force field for some of the less standard molecules such as $\mathrm{oEC}^{-}\bullet$ and CO_3^{2-} cannot be parametrized by SwissParam webserver.[3] These molecules cannot be parametrized by LigParGen force field webserver either.[4] We input oEC^{2-} SMILES as the input to LigParGen. To comply with the LigParGen-generated *.lmp file force field styles, the bond and angle styles must be LAMMPS harmonic style, the dihedral style must be LAMMPS opls style, and the improper style must be LAMMPS cvff style. We then modify the parameters corresponding to the radical carbon so that it prefers the planar trigonal sp^2 geometry instead of the tetrahedral sp^3 geometry (**Supplementary Table 1**). See the figure below for comparison between oEC^{2-} and $\mathrm{oEC}^{-}\bullet$ radical molecule geometries. We also extract relevant force field terms from LigParGen oEC^{2-} force field and from prior work on alkaline-earth carbonates[5] to construct an approximate CO_3^{2-} force field which prefers its planar trigonal sp^2 geometry (**Supplementary Table 2**).

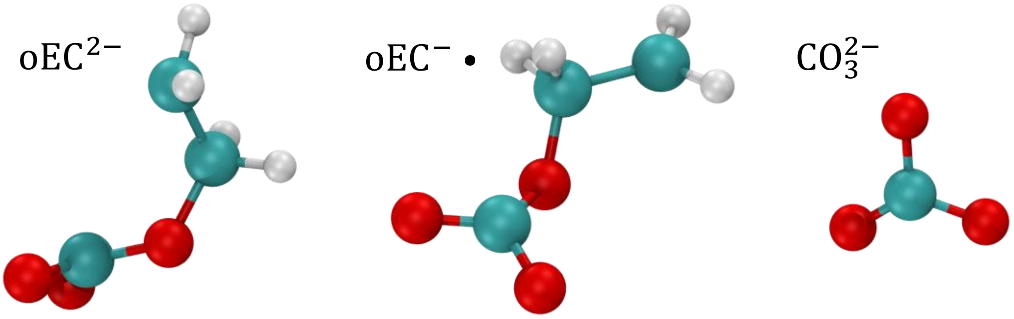

Supplementary Figure 6 | The molecule geometries produced by 3T-FF using oEC^{-2}, $\mathrm{oEC}^{-}\bullet$, and CO_3^{2-} force fields. The oEC^{-2} geometry is produced by LigParGen force field and is different from the radical we obtained at the end of 3T-VASP electrochemical reduction reactions. We modify the force field parameters so that we still have $\mathrm{oEC}^{-}\bullet$ radical geometry after the end of 3T-FF during the electrochemical oxidation reaction simulations.

<table>
<tr>
<td>oEC-1 LigParGen LAMMPS data

10 atoms
9 bonds
13 angles
11 dihedrals
4 impropers
10 atom types
9 bond types
13 angle types
11 dihedral types
4 improper types
-0.266390 49.733610 xlo xhi
-0.453290 49.546710 ylo yhi
-4.110460 45.889540 zlo zhi

Masses

1 12.011
2 15.999
3 15.999
4 12.011
5 12.011
6 1.008
7 1.008
8 15.999
9 1.008
10 1.008

Pair Coeffs

1 0.070 3.5500000
2 0.210 2.9600000
3 0.140 2.9000000
4 0.066 3.5000000
5 0.076 3.5500000
6 0.030 2.5000000
7 0.030 2.5000000
8 0.210 2.9600000
9 0.030 2.5000000
10 0.030 2.5000000

Bond Coeffs

1 656.0000 1.2500
2 214.0000 1.3270
3 320.0000 1.4100
4 317.0000 1.5100
~~5 340.0000 1.0800~~
~~6 340.0000 1.0800~~
5 372.0660 1.0830
6 372.0660 1.0830
7 656.0000 1.2500
8 340.0000 1.0900
9 340.0000 1.0900</td>
<td>Angle Coeffs

1 83.000 123.400
2 83.000 116.900
3 50.000 109.500
~~4 35.000 117.000~~
~~5 35.000 117.000~~
4 38.501 121.004
5 38.501 121.004
6 80.000 126.000
7 35.000 109.500
8 35.000 109.500
9 35.000 109.500
~~10 35.000 117.000~~
10 26.267 119.523
11 33.000 107.800
12 83.000 123.400
13 35.000 109.500

Dihedral Coeffs

1 4.669 5.124 0.000 0.000
2 -1.220 -0.126 0.422 0.000
3 0.000 0.000 ~~0.468~~ 0.000 0.000
4 0.000 0.000 ~~0.318~~ 0.000 0.000
5 0.000 0.000 ~~0.318~~ 0.000 0.000
6 0.000 0.000 0.198 0.000
7 0.000 0.000 0.198 0.000
8 4.669 5.124 0.000 0.000
9 0.000 0.000 ~~0.468~~ 0.000 0.000
10 0.000 0.000 ~~0.318~~ 0.000 0.000
11 0.000 0.000 ~~0.318~~ 0.000 0.000

Improper Coeffs

1 ~~2.500~~ 10.500 -1 2
2 10.500 -1 2
3 0.000 -1 2
4 0.000 -1 2

Atoms

1 1 1 0.50320000 1.000 1.00000 0.00000
2 1 2 -0.68290000 -0.266 1.00000 0.00000
3 1 3 -0.37510000 1.648 1.00000 -1.18280
4 1 4 0.19210000 0.944 0.40274 -2.25071
5 1 5 -0.95140000 0.860 1.38859 -3.36347
6 1 6 0.02990000 1.602 2.17268 -3.43992
7 1 7 0.02990000 0.087 1.28939 -4.11046
8 1 8 -0.68290000 1.754 1.00128 1.01549
9 1 9 -0.03150000 -0.052 0.02017 -2.00721
10 1 10 -0.03150000 1.536 -0.45329 -2.59359</td>
<td>Bonds

1 1 2 1
2 2 3 1
3 3 4 3
4 4 5 4
5 5 6 5
6 6 7 5
7 7 8 1
8 8 9 4
9 9 10 4

Angles

1 1 2 1 3
2 2 1 3 4
3 3 3 4 5
4 4 4 5 6
5 5 4 5 7
6 6 2 1 8
7 7 3 4 9
8 8 3 4 10
9 9 5 4 9
10 10 6 5 7
11 11 9 4 10
12 12 3 1 8
13 13 5 4 10

Dihedrals

1 1 4 3 1 2
2 2 5 4 3 1
3 3 6 5 4 3
4 4 10 4 5 6
5 5 9 4 5 7
6 6 9 4 3 1
7 7 10 4 3 1
8 8 8 1 3 4
9 9 7 5 4 3
10 10 10 4 5 7
11 11 9 4 5 6

Impropers

1 1 5 4 6 7
2 2 1 2 3 8
3 3 4 9 3 5
4 4 4 10 3 5</td>
</tr>
</table>

Supplementary Table 1 | 3T-FF classical force field parameters for $oEC^{-}\bullet$ radical. Neither LigParGen nor SwissParam can parametrize $oEC^{-}\bullet$ radical force field. We input oEC^{2-} SMILES as the input to LigParGen. We then modify the parameters corresponding to the radical carbon so that it prefers the planar trigonal sp^2 geometry instead of the tetrahedral sp^3 geometry. The relevant force field parameter modifications are highlighted in red. oEC^{2-} atom coordinates are unchanged because 3T-FF will immediately correct the geometry into the desired $oEC^{-}\bullet$ geometry. This force field is designed for 3T-FF usage (which will immediately be followed by a more robust 3T-VASP) and should not be used in a classical MD.

```
CO3-2 LigParGen LAMMPS data

4 atoms
3 bonds
3 angles
0 dihedrals
1 impropers
4 atom types
3 bond types
3 angle types
0 dihedral types
1 improper types
-0.266390    49.733610 xlo xhi
-0.453290    49.546710 ylo yhi
-4.110460    45.889540 zlo zhi

Masses

1    12.011
2    15.999
3    15.999
4    15.999

Pair Coeffs

1     0.070  3.5500000
2     0.190  2.9400000
3     0.190  2.9400000
4     0.190  2.9400000

Bond Coeffs

1   361.0000     1.3042
2   361.0000     1.3042
3   361.0000     1.3042
```

```
Angle Coeffs

1     83.000    120.000
2     83.000    120.000
3     83.000    120.000

Dihedral Coeffs


Improper Coeffs

1     10.500     -1      2

Atoms

1     1     1 0.50320000    1.000  1.00000  0.00000
2     1     2 -0.58030000   -0.266  1.00000  0.00000
3     1     3 -0.58030000    1.648  1.00000 -1.18280
4     1     4 -0.58030000    1.754  1.00128  1.01549

Bonds

1     1     1     2
2     2     1     3
3     3     1     4

Angles

1     1     2     1     3
2     2     2     1     4
3     3     3     1     4

Dihedrals


Impropers

1     1     1     2     3     4
```

Supplementary Table 2 | 3T-FF classical force field parameters for CO_3^{2-}. This force field is designed for 3T-FF usage (which will immediately be followed by a more robust 3T-VASP) and should not be used in a classical MD.

Impact of 3T Cycle Transition

The transition between 3T cycles re-initializes the 3T structure optimization problem into a new 3T parameter hyperspace, despite the continuous structure geometry trajectory (see main text **Methods**). This helps the structure to quickly escape local energy minimum in the old 3T parameter hyperspace. Visually, this manifests most strongly during the 3T-FF structure relaxation of the bulk electrolyte liquid molecules, where a structure which no longer undergoes much additional optimization experiences significantly larger optimization gradients and faster energy minimization. Note that in later cycle transitions, the 3T-FF structures' energies have settled and the small energy spikes (corresponding to small structure distortion induced by the Adam optimizer optimization) quickly return to their baseline energy levels.

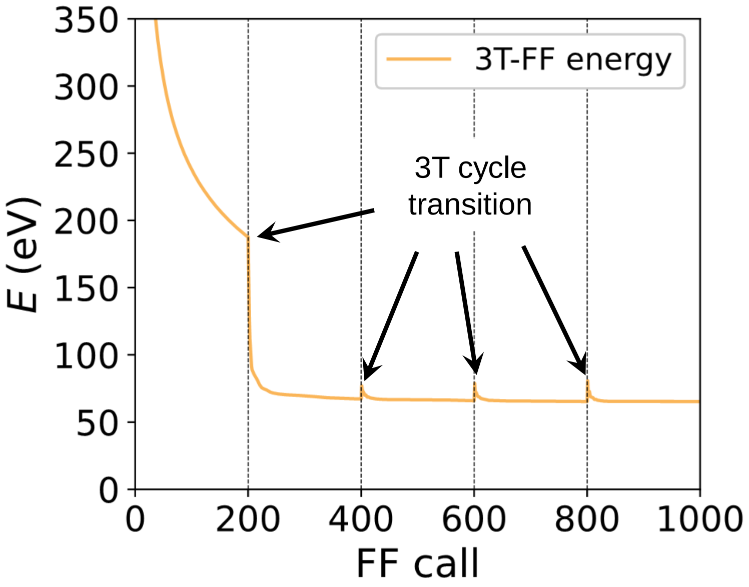

Supplementary Figure 7 | Example of 3T-FF potential energy of bulk liquid electrolyte through 3T cycle transitions. 5 cycles × 200 steps are used during this 3T-FF process, with initial molecules' structure at step 0 being packed by PackMol.[2] Source data are provided as a Source Data file.

Automated Identification of 3T-VASP Reactions

The chemical reaction in a multi-molecular system can be regarded as the recombination of the atoms in the molecules, which allows us to use some graph algorithms to help us automatically identify chemical reactions. The process of automatic reaction identification is mainly based on the connected component recognition algorithm and the isomorphism testing algorithm of graph. Its workflow sequence is listed below:

1. The molecular graph of the whole system is constructed based on the positions of the atoms, in which the connection relationship is determined based on 1.20 times the covalent bonding distance as the cut-off value.
2. The entire system is divided into different molecular groups through the connected component recognition algorithm of the graph.
3. Identify changes in molecular structures by comparing and analyzing the difference between the molecular group components of the current system and the initial state. Molecular groups that meet the following three conditions are classified as being involved in chemical reactions:
    a. The atomic indices contained in the molecular group change.
    b. The atomic indices are the same, but the current structure is not isomorphic to its initial state.
    c. The molecular group is isomorphic to the initial state, but the key geometric features are inconsistent.
4. After the above three steps, we can screen out molecules that do not participate in the reaction by aligning the atomic indexes, track and obtain the geometric structures of the reactants and products participating in the same reaction.

In this paper, we selected the improper angles between the carbonyl oxygen and the ring in the carbonate group of EC, DMC and VC, and between the alkene hydrogen atom and the ring in VC as the key geometric features for step 3c above. Molecules will be marked as participating in a charge

reduction/oxidation event if the corresponding improper angle is shifted by more than 15 degrees compared to the reactants.

By performing a similar analysis on the system structure of each frame obtained by the 3T algorithm, we can track the change process of the reactants. Taking the final reaction state as a reference, we can use difference comparisons to track when the reaction started and how it was transformed into the final product step by step throughout the calculation process.

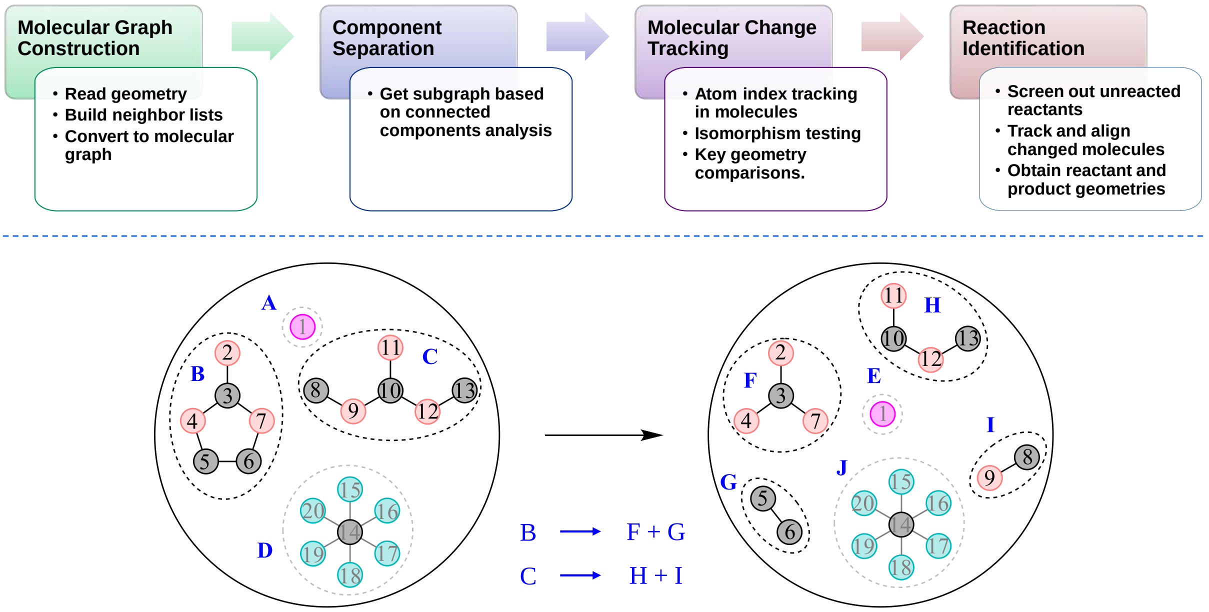

Supplementary Figure 8 | Workflow for automatic identification of 3T-VASP reactions. The chemical reaction in the multi-molecular system is regarded as the recombination of atoms in the molecule, and the chemical reaction is automatically identified by analyzing and tracking the change of the atomic index in each molecular group in the system through the graph algorithm. An example is shown at the bottom, after this workflow, we can get two reactions involving B and C respectively.

The procedure above produces a significant number of electrochemical reduction reactions and charge reduction events. However, some of these events are transient in nature, which are caused by 3T-VASP cycle transition every 50 DFT calls (see the previous section). During the first few steps of this transition, the structures have sufficient $\vec{\boldsymbol{\theta}}_t$ gradient (and correspondingly $\Delta\vec{\boldsymbol{\theta}}_t$ and $\Delta\vec{\mathbf{r}}_t$), which enable the molecules to temporarily climb the potential energy landscape and enter higher-energy states, before quickly relaxing into the initial or other lower energy states. When the molecules undergo these events but quickly return to their initial states, we consider these transient events which should not

be counted together with the real electrochemical reactions and charge reduction/oxidation events (main text). These transient events, separated by the corresponding input molecules are plotted below.

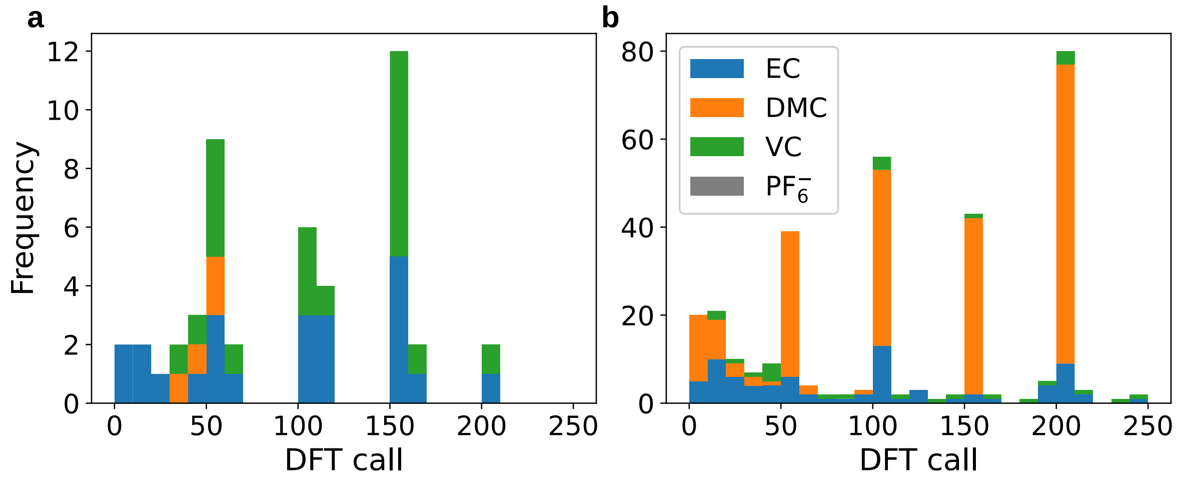

Supplementary Figure 9 | Histogram of transient 3T-VASP events. **a)** Transient electrochemical reduction reaction events. **b)** Transient electrochemical charge reduction events. These statistics are summed over 63 different trajectories, with a bin size of 10 DFT calls. These transient 3T-VASP events are separate from the real events plotted in main text **Figure 3**, and a majority occur within the first 10 DFT steps after the transitions into a new 3T-VASP cycle. Source data are provided as a Source Data file.

Electrolyte Electrochemical Reduction Reaction Events

We list all the reactions we have seen in our 63 reduction trajectories, where chemical bonds break (main text **Figure 3**) below:

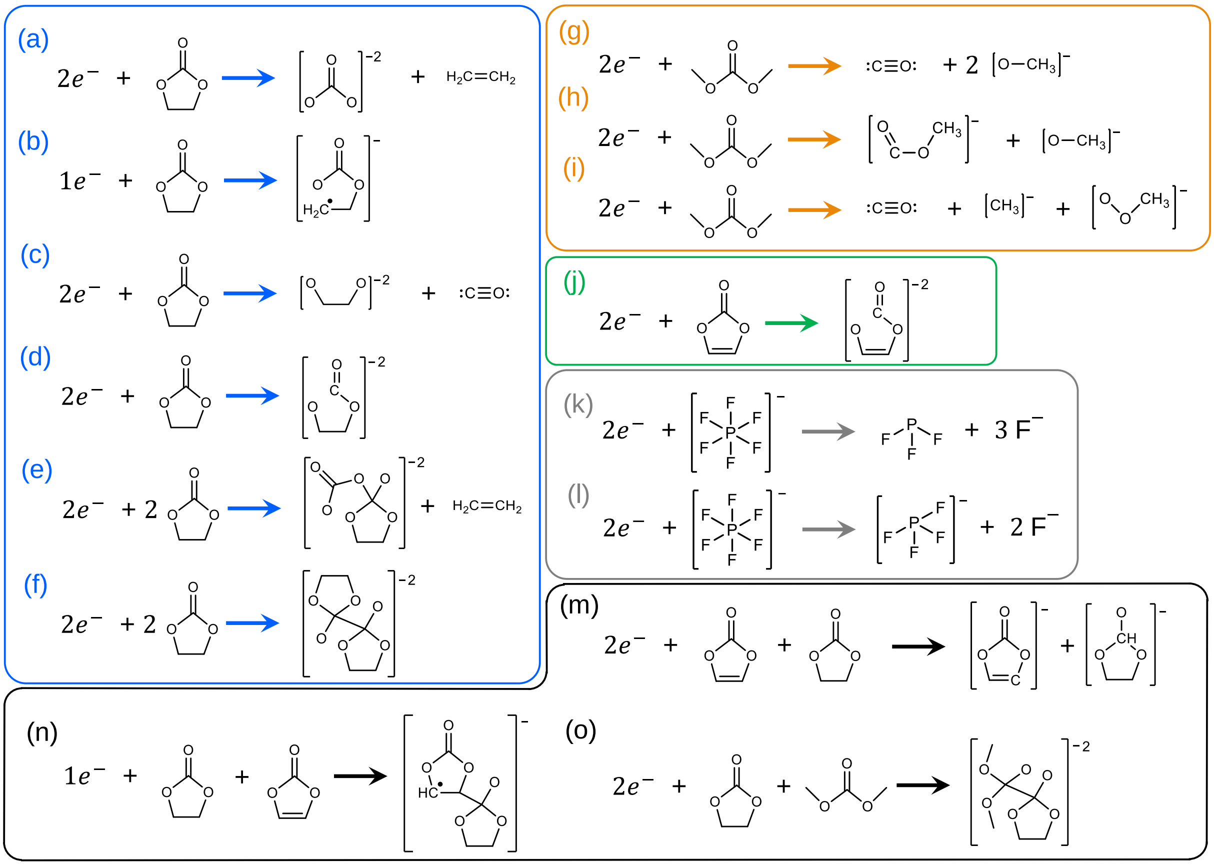

Supplementary Figure 10 | Electrolyte liquid electrochemical reduction reaction types observed using 3T-VASP. **a-f)** Reactions originating from EC molecules. **g-i)** Reactions originating from DMC molecules. **j)** Reactions originating from VC molecules. **k-l)** Reactions originating from PF_6^- anions. **m-o)** Cross-species reactions. EC -based reactions dominate electrochemical reduction reactions (main text **Figure 3b**). The lithium ions involved in these reactions are omitted.

Electrolyte Molecule Charge Reduction Events

We define electrolyte electrochemical charge reduction events as the situation where the electrolyte molecules are ionized into their [-1] state. In the 3T trajectory, these molecules are identified through their geometries because the improper angle change due to charge reduction involving the sp^2 carbon groups from the neutral molecules are obvious. These geometries are further confirmed to be the [-1] states of the molecules using quantum chemistry simulation, where the molecules maintain their twisted geometries. These reduction events require 1 electron, and do not happen for systems with $\mathrm{n_{excess}} = 0$. We found one reduction mode for the EC molecules, two distinct reduction modes for the DMC molecules, and three distinct reduction modes for the VC molecules. Quantum chemistry simulations confirm that all these identified modes are stable $\mathrm{EC^-}$, $\mathrm{DMC^-}$, and $\mathrm{VC^-}$ geometries.

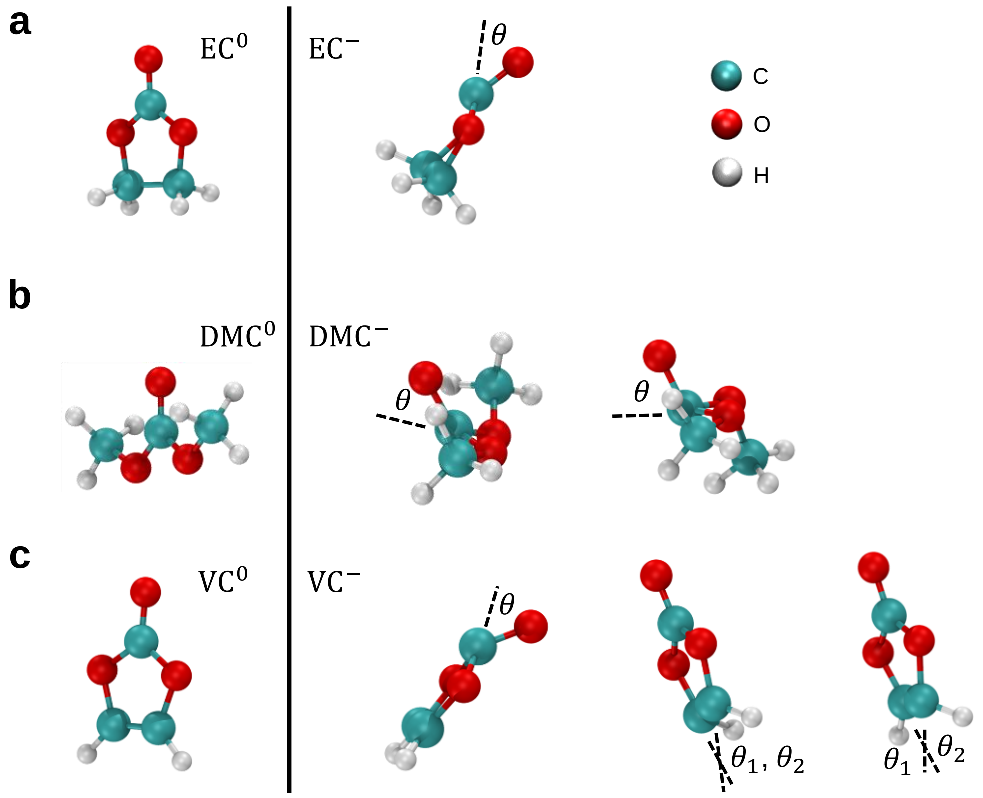

Supplementary Figure 11 | Electrolyte molecule charge reduction identification by geometry. a) $\mathrm{EC^-}$ geometry. **b)** $\mathrm{DMC^-}$ geometries (two modes: 1. both -CH_3 arms point up, 2. one arm up and one arm down). **c)** $\mathrm{VC^-}$ geometries (three modes: 1. bent O-C-O angle, 2. both H atoms are bent in the same direction, 3. both H atoms are bent in opposite directions). The angles θ are used for fast identification of the reduced electrolyte molecules.

Electrolyte Electrochemical Oxidation Reaction Events

We list all the reactions we have seen in our 63 oxidation trajectories, where chemical bonds break (main text **Figure 4**) due to the reaction between $\mathrm{oEC}^{-}\bullet$ radical and other compounds below:

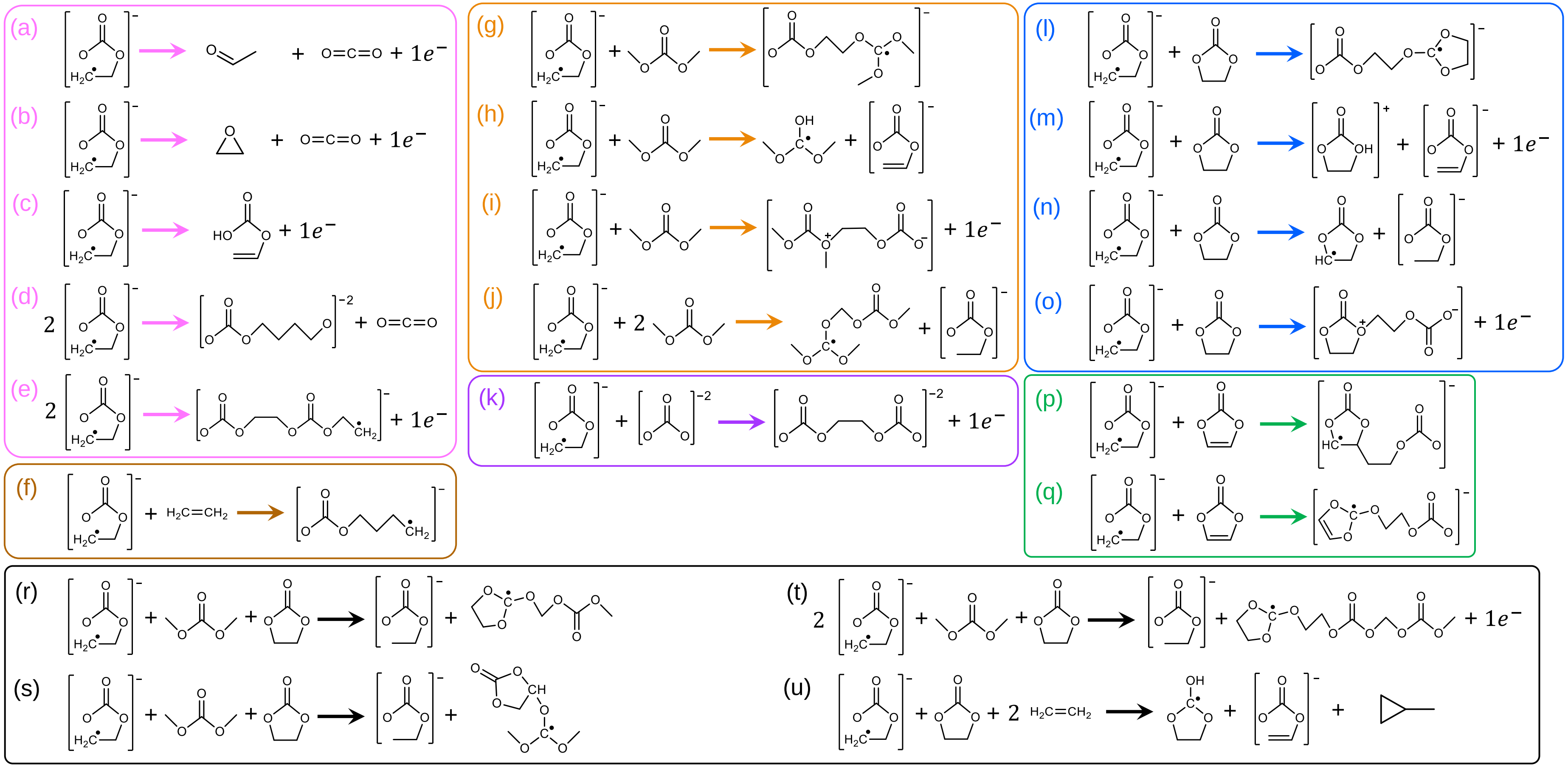

Supplementary Figure 12 | Electrolyte liquid electrochemical oxidation reaction types between oEC^{-} • and other compounds observed using 3T-VASP. a-e) Reactions originating purely from oEC^{-} • compound. Furthermore, we also observe reactions originating from oEC^{-} • reaction with other compounds: **f)** ethylene, **g-j)** DMC, **k)** CO_3^{2-}, **l-o)** EC, **p-q)** VC, **r-u)** two other species, forming long carbon chains. Reaction **(i)** and **(o)** form unstable compounds, likely because the oxidations are stopped after 150 DFT steps.

We also list all the observed oxidation reactions between CO_3^{2-} and other compounds:

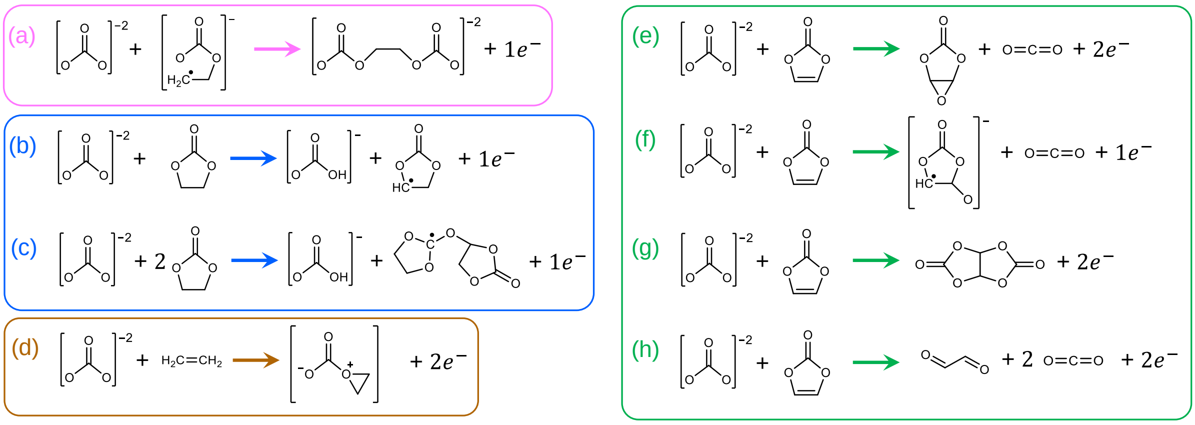

Supplementary Figure 13 | Electrolyte liquid electrochemical oxidation reaction types between CO_3^{2-} and other compounds observed using 3T-VASP. Reactions originating from CO_3^{2-} reaction with other compounds: **a)** $\mathrm{oEC}^{-}\bullet$, **b-c)** EC, **d)** ethylene, and **e-h)** VC. Reaction **(d)** forms an unstable compound, likely because the oxidation is stopped after 150 DFT steps.

Electrolyte Molecule Charge Oxidation Events

We define electrolyte electrochemical charge oxidation events as the situation where the electrolyte molecules are ionized into their [+1] state. In the 3T trajectory, it is difficult to automatically identify these molecules through just their geometries (unlike the charge reduction events) because the geometry changes are more subtle for EC and VC (although still relatively obvious for DMC). For EC and VC, they can primarily be identified by shorter C=O bond length. For DMC molecules, they can be identified using their sidechain bend angles in addition to the shorter C=O bond length. These geometries are listed below, and they are confirmed to be the [+1] states of the molecules using quantum chemistry simulation. We found one oxidation mode for the EC molecules, three distinct oxidation modes for the DMC molecules, and one oxidation mode for the VC molecules. Quantum chemistry simulations confirm that all these identified modes are stable EC^+, DMC^+, and VC^+ geometries.

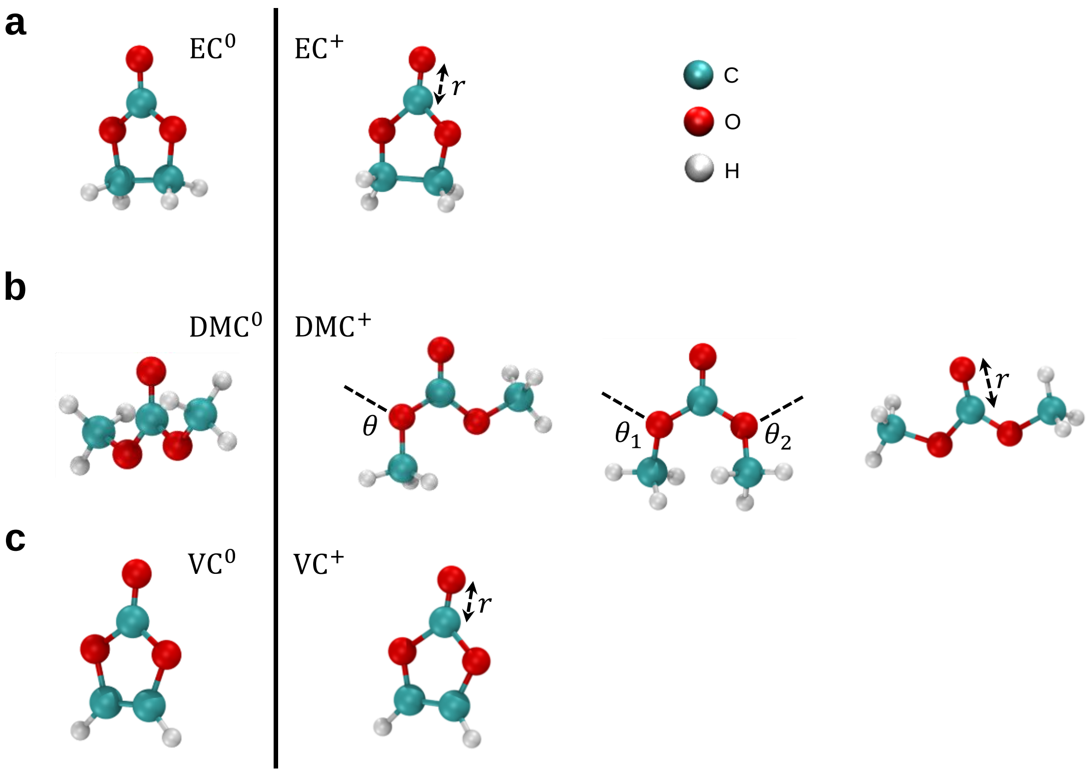

Supplementary Figure 14 | Electrolyte molecule charge oxidation identification by geometry. a) EC^+ geometry. **b)** DMC^+ geometries. **c)** VC^+ geometries. The angles θ can be used for fast identification of the oxidized DMC molecules, while the shorter C=O bond length can be utilized for the identification of the oxidized EC and VC molecules.

3T-VASP Electrochemical Reaction Byproduct Statistics

Reduction		Oxidation			
		$oEC^{-}\bullet$ in reactants		CO_3^{2-} in reactants	
Type	Count	Type	Count	Type	Count
a	60	a	30	a	4
b	4	b	17	b	1
c	1	c	4	c	1
d	6	d	1	d	1
e	1	e	1	e	4
f	2	f	1	f	1
g	1	g	2	g	1
h	3	h	1	h	1
i	2	i	1		
j	2	j	1		
k	1	k	4		
l	2	l	5		
m	1	m	1		
n	2	n	2		
o	1	o	1		
		p	1		
		q	1		
		r	2		
		s	1		
		t	1		
		u	1		

Supplementary Table 3 | 3T-VASP electrochemical reaction byproduct statistics for main text Figure 3 and Figure 4. These electrochemical reaction byproduct statistics are tabulated over 63 reduction and 63 oxidation 3T-VASP trajectories.

Lithium-Ion Battery Electrolyte–Anode Surface Reactions

We can also investigate liquid – solid surface electrochemical reactions instead of reactions within just the bulk liquid. These surface systems are typically bigger and slower to simulate, and we have not investigated them as thoroughly as we have for the bulk electrolyte liquid system. We list some of the reactions we have seen below, which are observed within 150–250 DFT steps using 3T energy relaxation. The surface we utilize is either lithium metal surface ($\mathrm{EC:DMC:VC} = 15:15:1$) or lithiated graphite <110> surface ($\mathrm{EC:DMC:VC} = 10:10:1$).

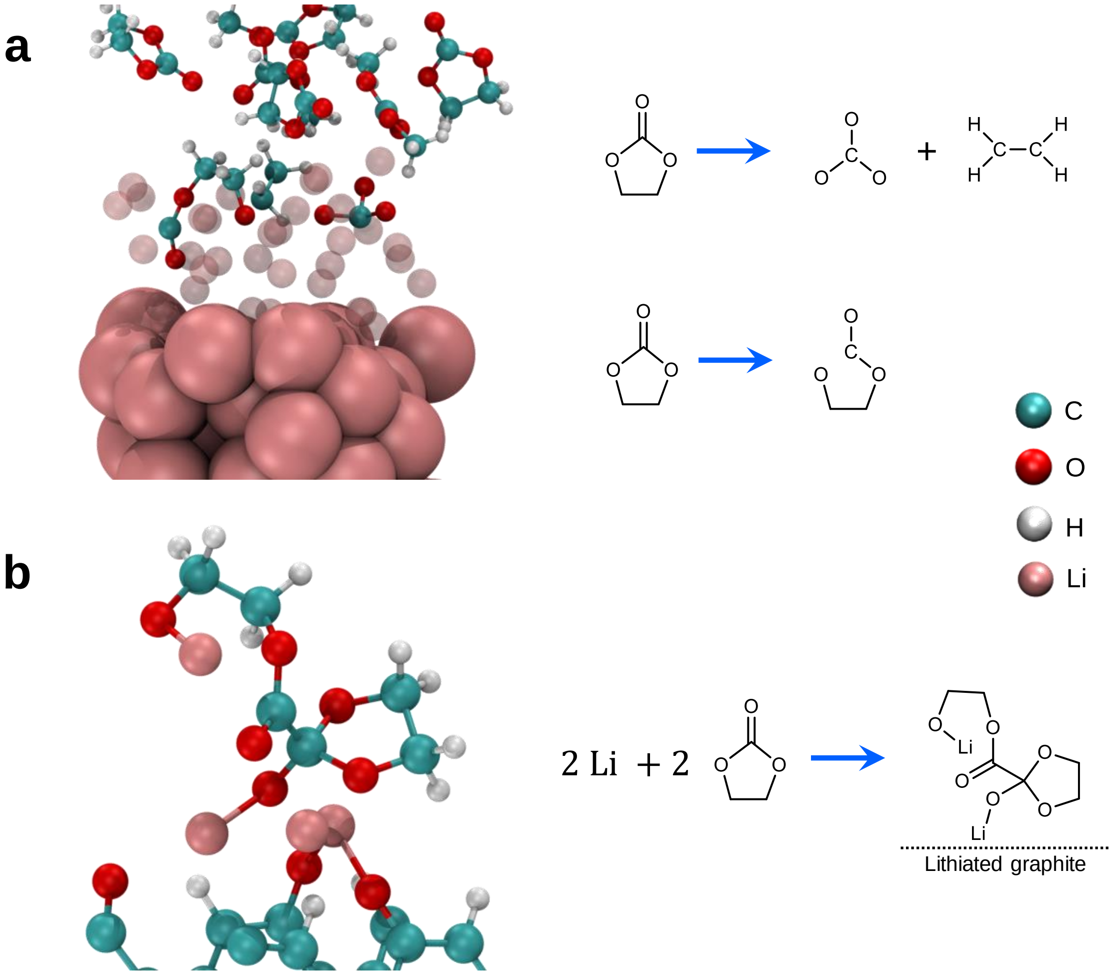

Supplementary Figure 15 | Electrolyte–anode surface reactions observed in 3T. a) Two separate EC molecules reacting on lithium metal surface. The EC molecules were able to react several layers deep within the lithium metal to form organolithium compounds, so the first few layers of lithium atoms have been made semi-transparent for clarity. While the reactions such as formation of carbonate and ethylene molecules or EC ring openings are typical, their geometries indicate that they no longer have sp^2 carbon atoms (and neither C=O nor C=C double bond remains). These reactions finish within three 3T-VASP cycles (3×50 = 150 DFT steps). **b)** Two different EC molecules reacting to form a long carbon chain on the edge of a lithiated graphite anode surface. This reaction requires two lithium atoms, with one lithium atom working to anchor the reacted EC molecule chain on the graphite surface (terminated with –O and –H atoms). Other non-reacting electrolyte molecules have been set invisible for clarity. These reactions finish within five 3T-VASP cycles (5×50 = 250 DFT steps).

Performance Comparison with VASP AIMD Baseline

We also attempt to quantify the speedup our 3T approach offers for the specific purpose of electrochemical reaction exploration, compared to an accelerated high-temperature AIMD approach. For this baseline, we use the same hardware we use for 3T energy minimization, but only utilize VASP AIMD at temperature T = 350 K. This temperature is approximately 50 K higher than the room temperature operation of the Li-ion battery, but still lower than the evaporation temperature of the electrolyte mixture liquid (DMC boils at T = 363 K). The system comprises of an oxidation reaction system with $\mathrm{n_{EC}}$ = 2, $\mathrm{n_{DMC}}$ = 10, $\mathrm{n_{VC}}$ = 2, $\mathrm{n_{C_2H_4}} = \mathrm{n_{CO_3^{2-}}}$ = 4, $\mathrm{n_{oEC^-\bullet}}$ = 4, $\mathrm{n_{Li^+}}$ = 5, and $\mathrm{n_{PF_6^-}}$ = 1. This system corresponds to $\mathrm{n_{excess}} = 2\mathrm{n_{CO_3^{2-}}} + \mathrm{n_{oEC^-\bullet}} + \mathrm{n_{PF_6^-}} - \mathrm{n_{Li^+}}$ = 8, which should be very oxidizing and contains a high concentration of reactive $\mathrm{oEC^-\bullet}$ radicals, with the aim of further accelerating chemical reaction events in the AIMD. Normal hydrogen mass of 1.008 AMU and timestep size of 0.5 fs are used, although we note that it is possible to double the timestep size by using the radioactive tritium hydrogen mass of 3.016 AMU instead of the standard hydrogen mass.[6] To ensure that the AIMD is focused on the electrochemical reactions and does not need to spend a long time on system equilibration, we utilize the last-step structure generated by 3T-FF as the initial structure for the AIMD (as opposed to starting AIMD from scratch using packmol molecule packing). The reactions we observe (plus the number of DFT steps and computation time) are shown in the figure below. In the AIMD, we observe relatively uniform reactions with three observed reactions transforming three different $\mathrm{oEC^-\bullet}$ radicals into $\mathrm{CO_2}$ and acetaldehyde. The fourth observed AIMD reaction involves a radical proton hopping from an $\mathrm{oEC^-\bullet}$ into an acetaldehyde through a DMC molecule intermediary. On the other hand, the observed 3T reactions happen earlier and are more diverse. The first reaction we observe is an EC dimerization reaction involving $\mathrm{oEC^-\bullet}$ and $\mathrm{CO_3^{2-}}$ to form ethylene dicarbonate anion ($\mathrm{EDC^{2-}}$), a commonly known electrochemical reaction byproduct of lithium-ion battery electrolytes.[7] The second reaction involves the decomposition of $\mathrm{oEC^-\bullet}$ into $\mathrm{CO_2}$ and ethylene oxide. The third reaction involves the transfer of F atom from $\mathrm{PF_6^-}$ to the $\mathrm{oEC^-\bullet}$, which produces an

oxyfluorocarbon compound and a PF_5^- radical (geometry confirmed to be stable via an MP2 quantum chemistry simulation) which will likely react further. The fourth reaction is the formation of peroxydicarbonate ($C_2O_6^{2-}$) anion which is known to be formed in highly oxidizing environments.[8] The fifth reaction involves $oEC^- \bullet$, ethylene, and DMC which forms a long hydrocarbon chain which is likely non-volatile (not observed among the gas byproducts of lithium battery electrolyte) and will participate in the formation of SEI network inside the battery electrolyte instead. We note that both AIMD and 3T trajectories here involve the usage of random seed, so re-runs will likely generate different trajectories and reactions at different DFT steps each time.

Surprisingly, in addition to being faster than VASP AIMD due to the smaller number of DFT steps needed to complete the electrochemical reactions (6× reduction in this specific case), 3T-VASP also benefits from shorter computation time needed for each DFT steps (a total of 19× reduction in this specific case). Further investigation indicates that this additional acceleration is caused by the VASP software CPU bottleneck during AIMD. For example, in one AIMD time step we observe VASP spending 75 s GPU computation time (presumably on a single self-consistent field, or SCF calculation) followed by 690 s CPU computation time (presumably on MD thermostat operation and other data management calculations). This contrasts with a total of 270 s computation time (GPU + CPU) spent for an observed 3T-VASP step. The significance of this CPU computation bottleneck depends on the system size (in larger systems the SCF GPU compute time may be the bottleneck) and hardware configuration (GPU-CPU vs just CPU), so we prefer to focus on the DFT step reduction and reaction diversity in this manuscript, which should be more generalizable across different material systems. We propose that ab-initio 3T energy minimization is useful for elementary electrochemical reaction exploration in complex material systems.

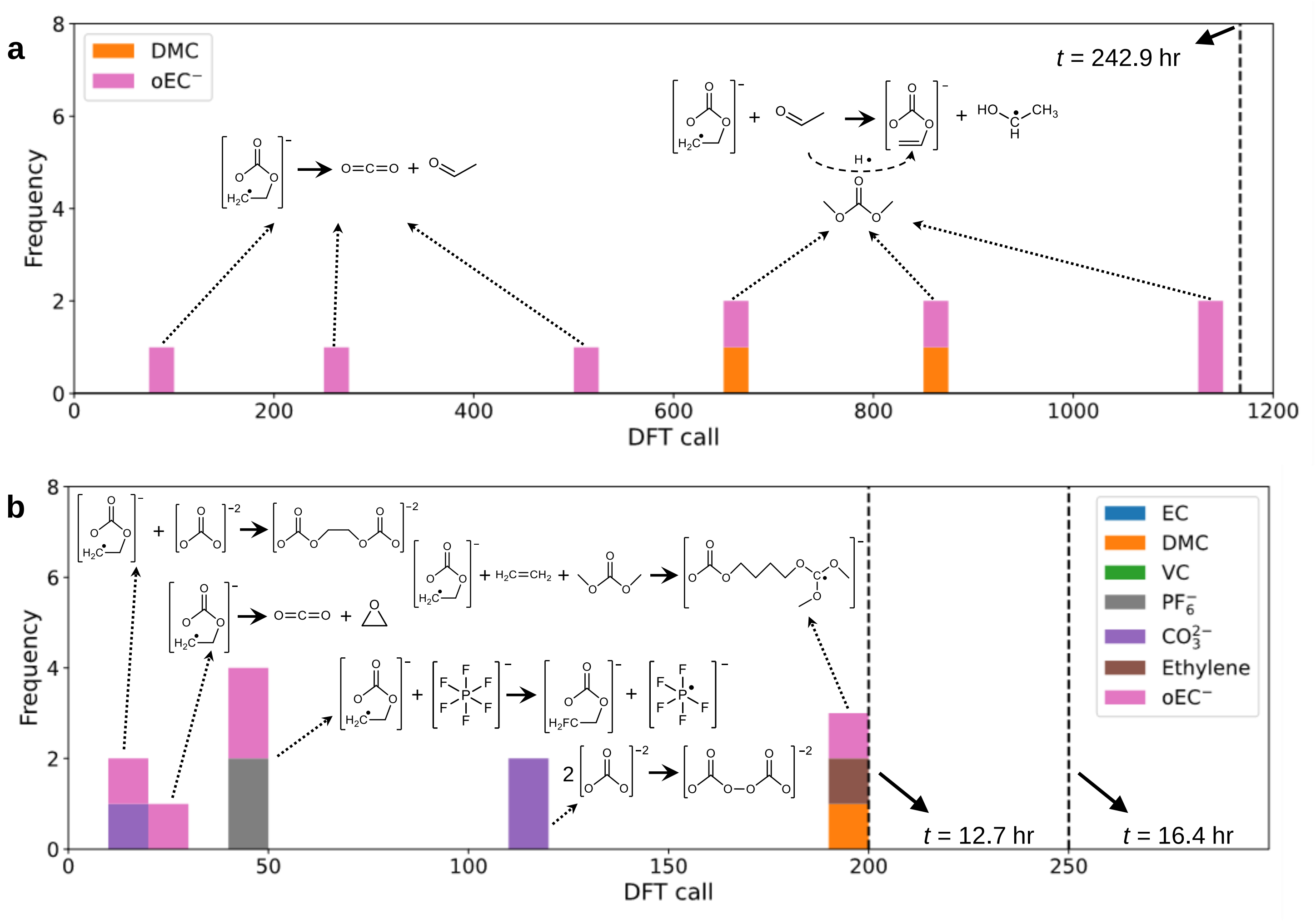

Supplementary Figure 16 | 3T-VASP trajectory comparison versus AIMD-VASP baseline. Highly oxidizing electrolyte conditions are used. **a)** Reactions generated in a 350K AIMD trajectory running for ~1200 steps (~600 fs, which is then stopped after 242.9 computation hours). The DMC only participates as an intermediary molecule for a radical proton hopping from $oEC^{-}\bullet$ to the acetaldehyde molecule. **b)** Reactions generated in 250 3T-VASP steps. For the last reaction, an intermediate reaction between the $oEC^{-}\bullet$ and ethylene molecule happens at DFT step 32 (not shown). All reactions are completed within 200 steps, which took a total of 12.7 computation hours. Source data are provided as a Source Data file.

Discussion on 3T Comparison with Molecular Optimization Internal Coordinate Methods

Our method shares a resemblance to the previously published internal coordinates method such as TRIC and its variants,[13,14] and less resemblance to that implemented by Sella.[15] In the following paragraphs, we compare 3T to these two methods.

TRIC freezes the internal coordinates of atoms within a defined sub-unit (for example the individual amino acids of a protein, or entire molecules) and then perform rotation / translation for the given sub-unit. This latter point on sub-structure rotation and translation is like 3T. TRIC employs a different representation for one level of sub-unit rotation/translation (using quaternion rotation matrix). While the math of the structure transformation is different, we believe that physically they will do a similar procedure of translating/rotating a set of atoms in a coordinated manner, which accelerates energy minimization. In the case of TRIC, the original Cartesian representation is reduced into a lower dimension set of internal coordinate representation.

However, the difference between 3T and TRIC method is that 3T is a multi-level structure transformation. In 3T, multiple level of hierarchical segmentations can be defined and the optimizations can be performed simultaneously. Unlike the TRIC method which needs to combine the translation and rotation into one layer quaternion matrix multiplication operation (and hence is meant to be performed on one level of user-defined sub-unit), 3T optimization is performed on multiple levels simultaneously (atom level, micro-group level which in our case corresponds to molecule fragments, and macro-group level which in our case corresponds to whole molecules). Hence, our method is not really an internal coordinate method because our parameter optimization hyperspace is of higher dimension than the initial Cartesian coordinate. The snapshot below from utils/run_utils.py describes our operation. The input tensor movable_pos_list is part of our optimization parameters (equivalent to optimizing per-atom T_{xyz} translation), in addition to parameters governing the other transformation operations.

```python
def create_optimizers(model, block):
    # We directly modify the atom xyz coordinates.
    # This is just a computation trick equivalent to modifying T_xyz, which saves a bit of compute/memory.
    theta_atom_translation = [param for param in model.movable_pos_list]
    optim_params = theta_atom_translation

    # Now we add the micro-groups' translation and rotation
    theta_micro_translation = model.translation_list
    theta_micro_rotation = model.rotation_list
    optim_params += [theta_micro_translation, theta_micro_rotation]

    special_rotation, macro_mode = model.special_rotation, model.macro_mode
    # Now we add sidechain micro-groups' rotatable bond axis rotation
    if special_rotation != None:
        theta_micro_axis_rotation = model.special_rotation_list
        optim_params += [theta_micro_axis_rotation]

    # Now we add macro-groups' translation and rotation
    if macro_mode != None:
        theta_macro_translation = model.macro_mode_translation_list
        theta_macro_rotation = model.macro_mode_rotation_list
        optim_params += [theta_macro_translation, theta_macro_rotation]

    optimizer = optim.Adam( optim_params , 3e-2, #1e-2,
                            weight_decay=0)
    optimizers = [ optimizer ]
    return optimizers
```

Because our method relies on the autograd capabilities developed by the machine learning community for high dimension neural network optimization, it is straightforward to add multiple levels of hierarchical segmentation and complex geometry transformation operations to be performed simultaneously, including those involving systems with periodic boundary conditions. One can simply compound new functions, as shown below in utils/potential_model_3T.py.

```python
    def arrange_atom_pos(self, movable_pos_list, fixed_pos):
        if self.special_rotation_idx != None:
            movable_pos_list = self.axis_rotate(movable_pos_list, fixed_pos)

        movable_pos_list = self.micro_rotate_translate(movable_pos_list)

        if self.macro_mode_idx != None:
            movable_pos_list = self.macro_rotate_translate(movable_pos_list)

        na = sum([movable_pos.shape[0] for movable_pos in movable_pos_list]) + fixed_pos.shape[0]
        atom_pos = torch.zeros(na,3).to(self.device)
        for i in range(len(movable_pos_list)):
            atom_pos[self.movable_idx_list[i],:] = movable_pos_list[i]
        atom_pos[self.fixed_idx,:] = fixed_pos

        return atom_pos
```

To the best of our understanding, if we turn off the 3T structure transformation operations per-atom T_{xyz} and micro-group A_m, R_m, T_m (hence we only allow one macro-group rotation R_M and

translation operation T_M each), turn off the capability for automatically managing proper intra-group coordinates over periodic boundary condition using PyTorch autograd, and remove the usage of PyTorch Adam optimizer to manage parameter stepping over the calculated gradients, then the 3T method will strongly resemble that of TRIC (physically at least, as the actual math operations are different). However, this version of 3T will be very different from the work we have presented in the manuscript, as it will no longer be a hierarchical structure optimization method.

We next discuss the comparison between 3T and the Sella optimizer. The Sella optimizer is interesting because it uses a different approach; it works in the basis of redundant internal coordinates (bonds, angles, and dihedrals extracted from the system, in addition to the dummy atoms added to mitigate invalid angle problems which commonly plague these internal coordinate methods). Constraints are added to avoid an increase in the dimensionality of the optimization problem due to the addition of dummy atoms (additional constraints may be added based on Sella user's chemical intuition), before a Hessian matrix for the system is approximated by iteratively diagonalizing it in the basis of the redundant internal coordinates. In this sense, the Sella optimizer is more like the TRIC method and unlike the 3T method which works in a higher dimension parameter space. 3T also does not project the system into a set of internal coordinates, as it simply uses additional 3T parameters $\vec{\boldsymbol{\theta}}$ to geometrically transform the atom positions in the original Cartesian coordinate space (and consequently by construction the 3T optimization system cannot go invalid).

In addition to that, the Sella optimizer can automatically determine the molecule connectivity graph, analyse if an angle is about to go invalid, and add dummy atoms to mitigate this problem on the fly. In contrast, the 3T-VASP as it is currently designed automatically determines its hierarchical segmentation by processing the individual input molecules using RDKit cheminformatics library and segmenting the molecule based on its rotatable bonds. In a way, our segmentation is more like TRIC's way of separating a material system into sub-units although in our case it is done for many molecules in a condensed phase periodic boundary system. The authors did mention that Sella may add

unnecessary constraints if multiple molecules exist in the system (forcing bond between molecules), and in this situation Sella will switch to TRIC's method instead. One potential extension to our work in the future is to implement a molecule reaction analyser which hierarchically re-segment molecules on the fly after chemical reaction has been detected in the system. This is currently not done in 3T-VASP.

The Sella optimizer approach has been demonstrated to work well compared to the default molecule optimizers used for different quantum chemistry software (Q-Chem, NWChem, etc) for molecules with up to 125 atoms. During the peer review, we have been made aware that the Sella optimizer also works with periodic boundary condition software such as VASP and Quantum Espresso, and in this aspect the Sella and 3T approaches are similar, even if the underlying math are different.

In this context, a Hessian-based approach does not seem to be appropriate for 3T, as 3T works in higher dimensional hyperspace instead of the lower-dimension internal coordinate space typically employed in TRIC and Sella. In addition to that, our primary target in this ab-initio reactor manuscript work is the VASP DFT software instead of quantum chemistry software like Gaussian and TeraChem. We routinely work with 237–325 atoms in the PBC box and attempting to build a Hessian matrix on the fly for 3T systems (>1000 parameters are typical) may mean significant optimization step overhead compared to the actual optimization steps we have now (in general 50-100 VASP DFT steps, or up to 150 steps, seem to be sufficient). The more commonly accepted approach in the neural network community when working in this high dimension parameter system and complex matrix operations is to leverage the computational efficiency of backpropagation autograd and the appropriate choice of parameter optimizer. In this case we choose Adam due to the many benefits it has in non-convex optimization problems[16] including requiring almost no hyperparameter tuning. In fact, all three examples we have shown in our work (perovskite, reduction, oxidation) use the same PyTorch Adam optimizer hyperparameter (just learning rate = 0.03, the other hyperparameters are left at PyTorch Adam default values), which we first decided on based on our observations during the perovskite energy minimization experiment. While Adam optimizer only requires first-order gradient, it adapts

individual adaptive learning rates for different parameters from estimates of first and second moments of the gradients.

In summary, we believe this is how our 3T approach differs from the internal coordinate methods TRIC and Sella:

1. TRIC and Sella optimize parameters in lower dimension internal coordinates ($< 3n_{\mathrm{atom}}$), while 3T optimize parameters in higher dimensions ($> 3n_{\mathrm{atom}}$).
2. TRIC constraints some relative coordinates between atoms (it is also an option available for Sella users), while 3T does not impose any such limitation (we still allow individual atom translation).
3. TRIC seems to be a single-level structure minimization (sub-unit scale defined in the software) which is to an extent a subset of 3T multi-level structure minimization, while Sella does single-level structure minimization in the redundant internal coordinate space (which extends from the bond scale up to the dihedral scale).
4. TRIC and Sella attempts to build a partial/approximate Hessian matrix on the fly to accelerate optimization in the reduced parameter space, while 3T relies on machine learning community's Adam optimizer to accelerate optimization in the enlarged parameter space. Adam optimizer is widely used for large-parameter neural network optimization.
5. TRIC, Sella, and 3T define the system parameter space in advance (internal coordinates for TRIC and Sella, and hierarchical transformation parameters for 3T), although Sella may also update its internal coordinates on the fly to facilitate the addition of new dummy atoms which is used to replace a previous internal coordinate which has gone invalid.
6. By construction, 3T parameter space does not go invalid because they are simply parameters which transform atom coordinates in the Cartesian space, unlike the internal coordinate methods where the Cartesian coordinates may go invalid when projected into the internal coordinate space (such as angles going to 0 or 180 degrees).

Discussion on 3T Comparison with Sella for Single Molecule Optimization

In this section, we briefly discuss about the advantage of 3T optimizer compared to other state-of-the-art optimizers typically used for expensive quantum chemistry-based single molecule optimization, such as Sella. We develop a simplified version of the 3T optimizer which works with the ASE calculator interface (NWChem ASE calculator[17] is demonstrated in this example). This is a direct comparison to the Sella optimizer which can work with NWChem ASE calculator. We then apply these optimizers on a TE4PBA cation structure freshly downloaded from PubChem (and hence the initial structure is energetically non-optimal). We optimize the geometry of TE4PBA using:

1. Sella optimizer utilizing NWChem
2. 3T optimizer utilizing NWChem
3. 3T optimizer using off-the-shelf organic force field (3×100 steps, negligible computation cost) followed by 3T utilizing NWChem

From the **Supplementary Figure 17** below, it is immediately clear that the 3T-based optimizer requires less NWChem calls to reach low-energy structure compared to the Sella optimizer, although all 3 methods reach the same optimized energy level and molecule structure at the end. 3T-FF-NWChem requires more NWChem calls than 3T-NWchem because 3T-FF initially generates a slightly less optimal initial conformation due to the usage of classical force field, but it still requires less NWChem calls than Sella-NWChem. No hyperparameter tuning is performed on the 3T optimizer, as we have simply used the default Adam optimizer learning rate (0.03) that we have used throughout the manuscript. We do note that we have not performed the rigorous infrastructure work which has been done by the Sella optimizer in their Python package (such as the i-PI socket protocol to directly communicate with the NWChem executable which is useful for computation overhead reduction), as NWChem single molecule quantum chemistry energy optimization has not been the focus of our manuscript. This infrastructure development, as well

as large-scale molecule optimization benchmarking work, can be done in the future work for a 3T manuscript focusing on single molecule quantum chemistry energy optimization.

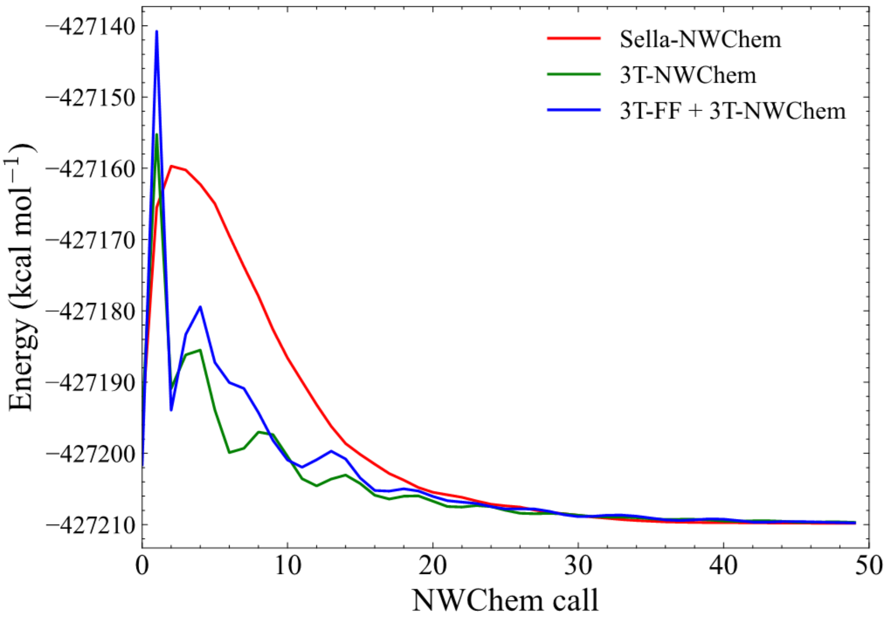

Supplementary Figure 17 | Comparison between Sella and 3T optimizers for single molecule energy optimization. In this example, we perform the energy optimization on the TE4PBA cation, which is a large and floppy organic cation consisting of 45 atoms. The ab-initio software being used by the optimizers is the quantum chemistry software NWChem. In the case of 3T-FF-NWChem, the structure is first optimized using 3T-FF for 3×100 steps (negligible computation cost) before starting 3T-NWChem energy minimization. 3T optimizers require less NWChem steps than the Sella optimizer to reach lower energy levels, although the final structures generated by Sella and 3T are equivalent in energy level and geometry. Source data are provided as a Source Data file.

Discussion on 3T Comparison with Sella for Bulk Electrolyte Electrochemical Reactions

In this final section, we compare the performance of 3T with the Sella optimizer for the primary task explored in this manuscript, which is the exploration of electrochemical reaction in bulk electrolyte. We attempt to understand the difference between 3T-VASP results compared to when Sella-VASP is utilized on the same system, under default Sella settings and identical cycle/step counts. We want to make sure that the Sella optimizer has access to the same kind of physically meaningful initial structure which has been produced by 3T-FF (so that Sella does not have to waste iteration steps dispersing molecules in the VASP periodic boundary condition box and can focus on the electrochemical reactions instead). Consequently, we take the FF structure we have previously generated using 5×200 steps of 3T-FF. This structure is converted into an ASE atoms object before being attached onto an ASE VASP calculator and passed into a Sella optimizer. We use the ASE calculator input settings for Sella-VASP which generate the same VASP input files (INCAR, KPOINTS, POTCAR) that are used for the 3T-VASP optimizations. We instruct Sella to work on optimization to an energy minimum (order=0), utilize its internal coordinates (internal=True), and run the structure energy minimization for 5×50 steps, just like 3T-VASP (to see if Sella can also benefit from inter-cycle transition like 3T-VASP). We do this for 3 randomly chosen 3T-FF reduction trajectories (**Supplementary Figure 18**), and for 3 randomly chosen 3T-FF oxidation trajectories (**Supplementary Figure 19**). These Sella-VASP trajectories are directly compared to the corresponding 3T-VASP trajectories which were generated from the same final 3T-FF structures. As a general summary, we observe the following trends:

1. Sella-VASP final structures always have higher energies than 3T-VASP final structures (0.63–13.46 eV difference observed)
2. 3T-VASP reactions are typically finished within less DFT calls compared to Sella-VASP reactions

3. 3T-VASP tends to generate the expected common reactions more frequently, while Sella-VASP has higher probabilities of generating more rare reactions. This is likely because Sella-VASP tends to end up with higher-energy structures compared to 3T-VASP. This has advantage (rare reaction exploration) and disadvantage (statistically less meaningful, unphysical/unfinished reactions), as we will later discuss.

4. Sella-VASP always utilizes more than the 5×50 DFT calls instructed to Sella. From our observation, this is likely related to the introduction/modification of dummy atoms throughout the Sella-VASP optimization.

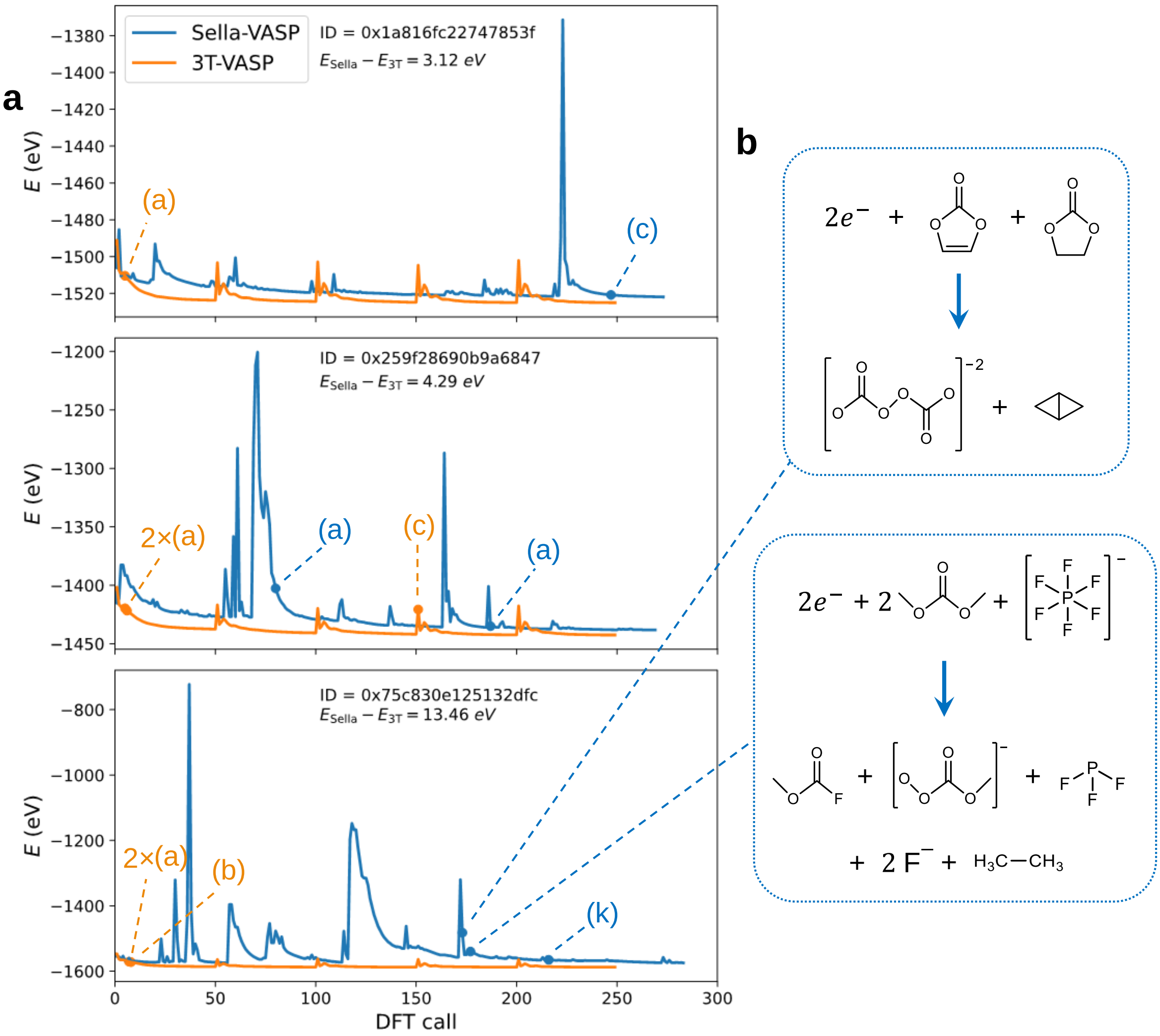

Supplementary Figure 18 | Comparison between Sella and 3T relaxations for VASP electrolyte reduction reaction exploration. (a) Comparison of the structure energy vs VASP call when 3 different 3T-FF final structures are used as the initial structures for relaxation. Please refer to **Supplementary Figure 10** for the exact reaction types we have annotated. **(b)** Novel reactions generated by Sella-VASP which have not been seen in prior 3T-VASP electrolyte reduction trajectories. Source data are provided as a Source Data file.

We first discuss the Sella-VASP electrolyte reduction experiment. From the **Supplementary Figure 18** above, we can see that the energy minimization profile curve of Sella-VASP is not very stable and settles at relatively high energy (3.12–13.46 eV higher) compared to the identical initial structure minimized using 3T-VASP. It seems that the current Sella-VASP bulk electrolyte reduction structure minimization in the periodic boundary condition box tends to get stuck in higher local energy minima, with occasional attempts to escape the minima which results in very high energy spikes before quickly settling back down. As of August 7, 2024, the version of Sella publicly available to us for usage from Github[15] generates very large energy spikes up to more than 200 eV larger compared to the final relaxed Sella-VASP energy values. This behaviour is somewhat different than the smooth Sella-NWChem energy minimization curve for single molecule (**Supplementary Figure 17**), and we are unsure whether this is an expected feature of Sella-VASP bulk energy minimization or not. These high energy structures may contribute to the generation of unphysical reactions. While some of the Sella-VASP reactions we observe are expected (they correspond to 3T-VASP reactions a, c, and k from **Supplementary Figure 10**), we also observe two concerning new reactions (observed in the trajectory ID=0x75c830e125132dfc on **Supplementary Figure 18b**, where Sella-VASP final structure energy is significantly higher (+13.46 eV) compared to the 3T-VASP final structure energy). The first reaction between EC and VC generates bicyclo[1.1.0]butane ($\mathrm{C_4H_6}$), which is a highly strained organic molecule that we think is an unlikely electrolyte electrochemical reduction reaction byproduct. The second reaction between 2 DMC and $\mathrm{PF_6^-}$ generates many reaction byproduct molecules, including $\mathrm{C_2H_6}$. According to experimental literature, $\mathrm{C_2H_6}$ should not be observed as an electrolyte reduction decomposition byproduct gas when $\mathrm{EC{:}\,DMC}$ electrolyte mixture is used.[18] In contrast, $\mathrm{C_2H_6}$ is never observed as a 3T-VASP byproduct reaction in our 63 electrolyte reduction trajectories, in line with experimental observation. We note that while reaction c and k are also observed in 3T-VASP, they are supposed to be very infrequent (**Supplementary Table 3**). However, these reactions are easily obtained by Sella-VASP within just 3 trajectories. This indicates that Sella-VASP can generate rare

reactions more frequently because it is often stuck at higher local energy minimums. This may be a Sella-VASP advantage when rare reaction exploration is desired. On the other hand, 3T-VASP tends to explore lower-energy structures.

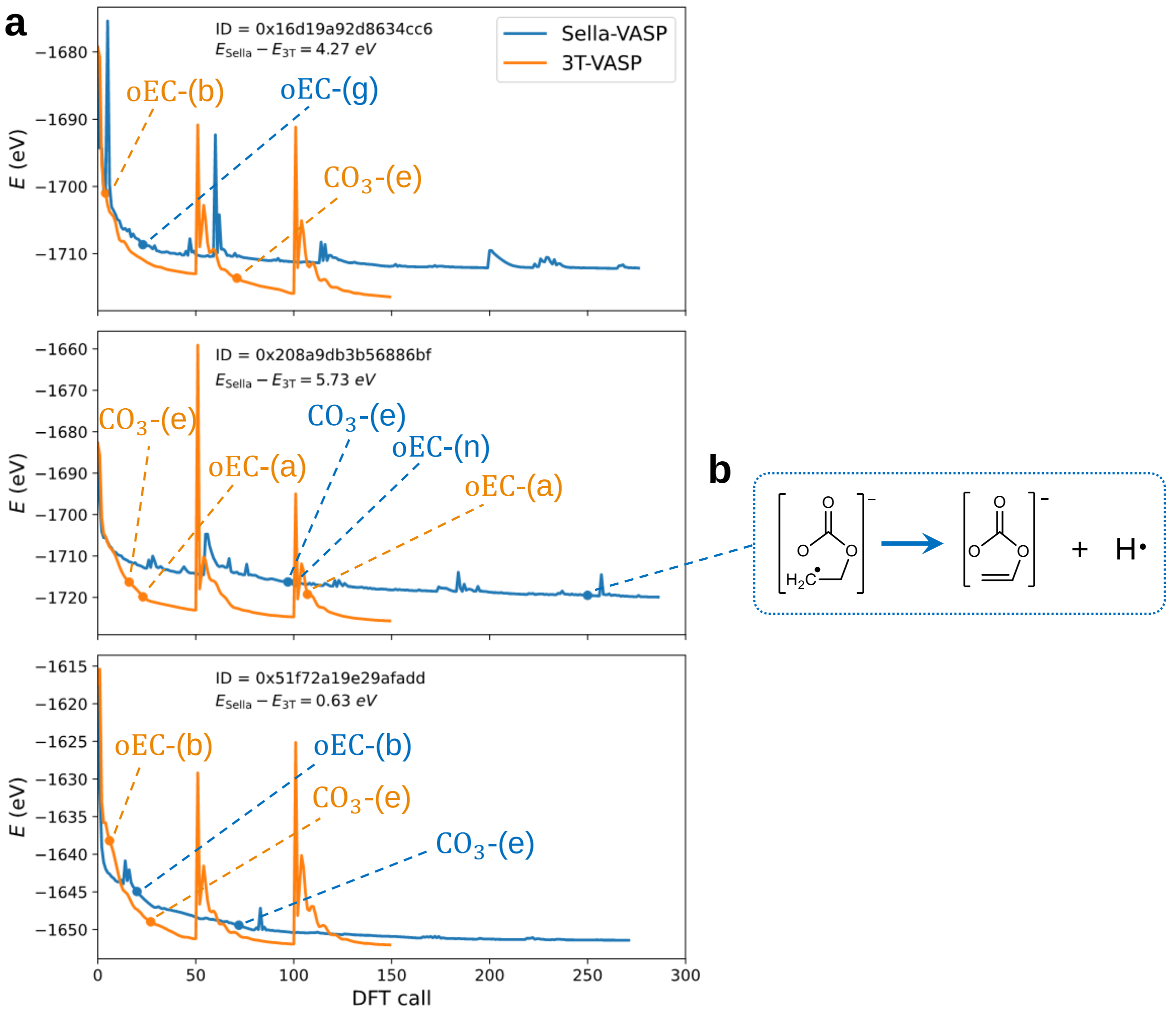

Supplementary Figure 19 | Comparison between Sella and 3T relaxations for VASP electrolyte oxidation reaction exploration. (a) Comparison of the structure energy vs VASP call when 3 different 3T-FF final structures are used as the initial structures for relaxation. Please refer to **Supplementary Figure 12** (oEC^{-} •) & **13** (CO_3^{2-}) for the exact reaction types we have annotated. **(b)** Novel reactions generated by Sella-VASP which have not been seen in prior 3T-VASP electrolyte oxidation trajectories. Source data are provided as a Source Data file.

However, the situation is very different for Sella-VASP oxidation reaction trajectories (**Supplementary Figure 19**). While the observed trend holds where Sella-VASP final structure energies are always higher than the corresponding 3T-VASP final structure energies (0.63–5.73 eV higher), we no longer observe the large (>200eV) energy spikes like the ones we observe in Sella-VASP electrolyte reduction trajectories. Sella-VASP still generates reactions more slowly than 3T-VASP, and just like

before it can find reactions which are less commonly encountered in 3T-VASP, such as oEC-(g) and oEC-(n) oxidation reactions (**Supplementary Figure 12**, **Supplementary Table 1**). However, we no longer observe unphysical reactions. The only reaction which looks somewhat concerning is an $\mathrm{oEC}^{-}\bullet$ decomposition reaction which produces a hydrogen radical. We have observed this structure before, which is a typical 3T-VASP intermediate byproduct which appears before an $\mathrm{oEC}^{-}\bullet$ containing reaction is finished. We believe that this is just an unfinished intermediate byproduct because Sella-VASP needs more steps before it can complete the reaction. We also note that the best-performing Sella-VASP energy minimization (ID=0x51f72a19e29afadd, where the final structure energy is only 0.63 eV above 3T-VASP) ends up generating identical reaction byproducts to the corresponding 3T-VASP version, although the reactions still occur more slowly compared to 3T-VASP.

This leads us to conclude that Sella-VASP, as currently available on August 7, 2024, is not very suitable for exploring electrochemical reduction reactions in bulk electrolyte using periodic boundary condition code such as VASP, as it generates relatively high energy structures which look less physical compared to 3T-VASP. For oxidation reactions, Sella-VASP is relatively competitive to 3T-VASP although it still requires more DFT calls to produce the desired reactions. When low-energy structure bulk electrolyte structure exploration is desired, 3T-VASP is more advantageous than Sella-VASP. However, if high-energy rare structure exploration is desired instead, Sella-VASP's tendency to be trapped in a higher local energy minimum may be advantageous compared to 3T-VASP (although more care is needed to ensure that the generated structures are physical).

Finally, we note that while Sella-VASP has been instructed to perform 5×50 steps of VASP energy minimizations, in practice it seems to automatically perform a few additional VASP calls in each cycle (making the total step count to be more than 250 steps). These extra VASP calls seem to happen when Sella ran into invalid angle internal coordinate problems and need to update the structure using new dummy atoms. This problem does not occur in 3T-VASP.

References

1. RDKit: Open-Source cheminformatics. http://www.rdkit.org/ (2020).
2. Martinez, L., Andrade, R., Birgin, E. G. & Martinez, J. M. PackMol: A package for building initial configurations for molecular dynamics simulations. *J. Comput. Chem.* **30**, 2157–2164 (2012).
3. Zoete, V., Cuendet, M. A., Grosdidier, A. & Michielin, O. SwissParam: A fast force field generation tool for small organic molecules. *J. Comput. Chem.* **32**, 2359–2368 (2012).
4. Dodda, L. S., De Vaca, I. C., Tirado-Rives, J. & Jorgensen, W. L. LigParGen web server: An automatic OPLS-AA parameter generator for organic ligands. *Nucleic Acids Res.* **45**, W331–W336 (2017).
5. Raiteri, P., Demichelis, R. & Gale, J. D. Thermodynamically consistent force field for molecular dynamics simulations of alkaline-earth carbonates and their aqueous speciation. *J. Phys. Chem. C* **119**, 24447–24458 (2015).
6. Leung, K. & Budzien, J. L. Ab initio molecular dynamics simulations of the initial stages of solid-electrolyte interphase formation on lithium ion battery graphitic anodes. *Phys. Chem. Chem. Phys.* **12**, 6583–6586 (2010).
7. Abbott, J. W. & Hanke, F. Kinetically corrected Monte Carlo-molecular dynamics simulations of solid electrolyte interphase growth. *J. Chem. Theory Comput.* **18**, 925–934 (2022).
8. Halilu, A., Hayyan, M., Aroua, M. K., Yusoff, R. & Hizaddin, H. F. In situ electrosynthesis of peroxydicarbonate anion in ionic liquid media using carbon dioxide/superoxide system. *ACS Appl. Mater. Interfaces* **11**, 25928–25939 (2019).
9. Pracht, P., Bohle, F. & Grimme, S. Automated exploration of the low-energy chemical space with fast quantum chemical methods. *Phys. Chem. Chem. Phys.* **22**, 7169–7192 (2020).
10. Grimme, S., Bannwarth, C. & Shushkov, P. A robust and accurate tight-binding quantum chemical method for structures, vibrational frequencies, and noncovalent interactions of large molecular systems parametrized for all spd-block elements (Z = 1-86). *J. Chem. Theory Comput.* **13**, 1989–2009 (2017).
11. Spicher, S. & Grimme, S. Robust atomistic modeling of materials, organometallic, and biochemical systems. *Angew. Chemie - Int. Ed.* **59**, 15665–15673 (2020).
12. Lu, T. Molclus, version 1.12, Beijing Kein Research Center for Natural Science. *http://www.keinsci.com/research/molclus.html, (accessed 2024-05-30)*.
13. Wang, L. P. & Song, C. Geometry optimization made simple with translation and rotation coordinates. *J. Chem. Phys.* **144**, (2016).
14. Shajan, A., Manathunga, M., Götz, A. W. & Merz, K. M. Geometry optimization: A comparison of different open-source geometry optimizers. *J. Chem. Theory Comput.* **19**, 7533–7541 (2023).
15. Hermes, E. D., Sargsyan, K., Najm, H. N. & Zádor, J. Sella, an open-source automation-friendly molecular saddle point optimizer. *J. Chem. Theory Comput.* **18**, 6974–6988 (2022).
16. Kingma, D. P. & Ba, J. L. Adam: A method for stochastic optimization. in *Proceedings of the 3rd International Conference on Learning Representations* 1–15 (2015).
17. Aprà, E. *et al.* NWChem: Past, present, and future. *J. Chem. Phys.* **152**, 184102 (2020).

18. Teng, X. *et al.* In situ analysis of gas generation in lithium-ion batteries with different carbonate-based electrolytes. *ACS Appl. Mater. Interfaces* **7**, 22751–22755 (2015).
